# Two new lanostanoid glycosides isolated from a Kenyan polypore *Fomitopsis carnea*

**DOI:** 10.3762/bjoc.19.84

**Published:** 2023-08-02

**Authors:** Winnie Chemutai Sum, Sherif S Ebada, Didsanutda Gonkhom, Cony Decock, Rémy Bertrand Teponno, Josphat Clement Matasyoh, Marc Stadler

**Affiliations:** 1 Department of Microbial Drugs, Helmholtz Centre for Infection Research GmbH (HZI), Inhoffenstraße 7, 38124 Braunschweig, Germany; 2 Institute of Microbiology, Technische Universität Braunschweig, Spielmannstraße 7, 38106 Braunschweig, Germany; 3 Department of Pharmacognosy, Faculty of Pharmacy, Ain Shams University, 11566 Cairo, Egypt; 4 Center of Excellence in Fungal Research, School of Science, Mae Fah Luang University, 333 Muang, Chiang Rai, 57100 Thailand; 5 Mycothéque de l’ Universite Catholique de Louvain (BCCM/MUCL), Place Croix du Sud 3, B-1348 Louvain-la-Neuve, Belgium; 6 Department of Chemistry, Faculty of Science, University of Dschang, P. O. Box 67, Dschang, Cameroon; 7 Department of Chemistry, Egerton University, P.O. Box 536, 20115, Njoro, Kenya

**Keywords:** antimicrobial activity, *Fomitopsis carnea*, lanostane glycosides, Polyporales

## Abstract

Chemical exploration of solid-state cultures of the polypore *Fomitopsis carnea* afforded two new C31 lanostane-type triterpenoid glycosides, forpiniosides B (**1**) and C (**2**) together with two known derivatives, namely 3-epipachymic acid (**3**) and (3α,25*S*)-3-*O*-malonyl-23-oxolanost-8,24(31)-dien-26-oic acid (**4**). The structures of the isolated compounds were established based on HRESIMS and extensive 1D and 2D NMR experiments. All the isolated compounds were assessed for their antimicrobial and cytotoxic activities. Among the tested compounds, forpinioside B (**1**) exhibited significant antimicrobial activity against *Staphylococcus aureus* and *Bacillus subtilis* at MIC values comparable to gentamycin and oxytetracycline (positive controls), respectively.

## Introduction

Great success was realized on antibiotic discovery between 1930 and 1960 during the ‘golden era’ of antibiotics. Unfortunately, the pace of antibiotic research and development in the face of emerging resistant pathogens has not been kept up, thus raising a big concern for a return to the pre-antibiotic era [1–2]. Recently, the danger posed by previously treatable microbial diseases has been increased with the emergence of ‘superbugs’. If not properly addressed, these circumstances will inevitably lead to an increase in healthcare-associated costs due to longer and more frequent hospital stays as well as to the need of multi-drug therapy [3].

Due to their well-known profuse production of bioactive molecules, Basidiomycota have already been proven to be a valuable source for new anti-infectives [4]. These include a myriad of triterpenoids which have been resourceful in the discovery of potent antimicrobials [5]. Specifically, lanostane triterpenoids are typical bioactive chemical constituents of various Polyporales. For instance, these compounds play a major role in the pharmacological effects of *Ganoderma* such as *Ganoderma lingzhi* (often incorrectly referred by some scientists as "*G. lucidum*"), a medicinal fungus widely used in traditional Chinese medicine (TCM) [6]. Studies on other *Ganoderma* species have also reported antidiabetic [7] and cytotoxic [8] effects of these compounds. Another widely used oriental medicine polypore *Wolfiporia* (also referred to under the synonym ‘*Poria*’ in the literature) *cocos*, has proved to a beneficial source of bioactive lanostane triterpenoids [9–10]. In addition, the edible European fungus *Macrolepiota procera* also produced antiproliferative and anti-inflammatory lanostanoid derivatives [11]. Furthermore, triterpenes from *Tricholoma pardinum* and *Fomitopsis betulina* (previously known as *Piptoporus betulinus*) exhibited nitric oxide (NO) and/or cytotoxic effects, respectively [12–13].

The genus *Fomitopsis* was first coined by Karsten typified by *F. pinicola* [14]. Our current study fungus *F. carnea* was reported for the first time from Japan in 1943 by Blume and Nees [15]. Later, Ryvarden and Johansen in 1980 [16] and Carranza-Morse and Gilbertson in 1986 [17] confirmed the presence of the species in Tanzania apart from Japan. Ortiz-Santana et al. [18] indicated the close relationship of the genus *Fomitopsis* to *Antrodia*. This led to more recent revisions on the genera based on multiple genes (nLSU, ITS, nSSU, mtSSU, rbb2 and tef1), placing *F. carnea* in the *Rhodofomes* clade [15].

The genus *Fomitopsis* has proven to be an invaluable source of bioactive molecules [18–23]. Fruit body infusions of *F. betulina* have been widely used in folk medicine in combating various diseases and ailments [20]. Chemical investigations of *F. pinicola* or *F. betulina* revealed their cytotoxic [21,23] and antidiabetic propensities [19]. In addition, the recent review on *F. officinalis*, elaborated the potential use of its compounds as antibiotic leads [19].

**Figure 1:**
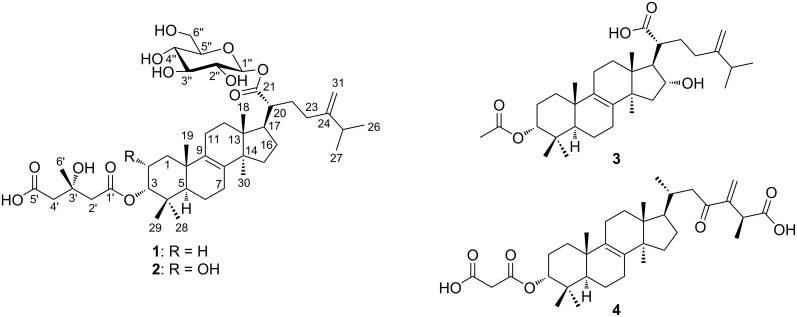
Chemical structures of compounds **1**–**4**.

In this study we report the isolation and structure elucidation of two new C31 lanostane-type triterpenoid glycosides (compounds **1** and **2** in Figure 1) together with two known derivatives, namely 3-epipachymic acid (3α-acetoxy-16α-hydroxy-5α-lanost-8,24(31)-dien-21-oic acid (**3**)) [24] and (3α,25*S*)-3-*O*-malonyl-23-oxolanost-8,24(31)-dien-26-oic acid (**4**) [25].

## Results

### Structure elucidation

**Figure 2:**
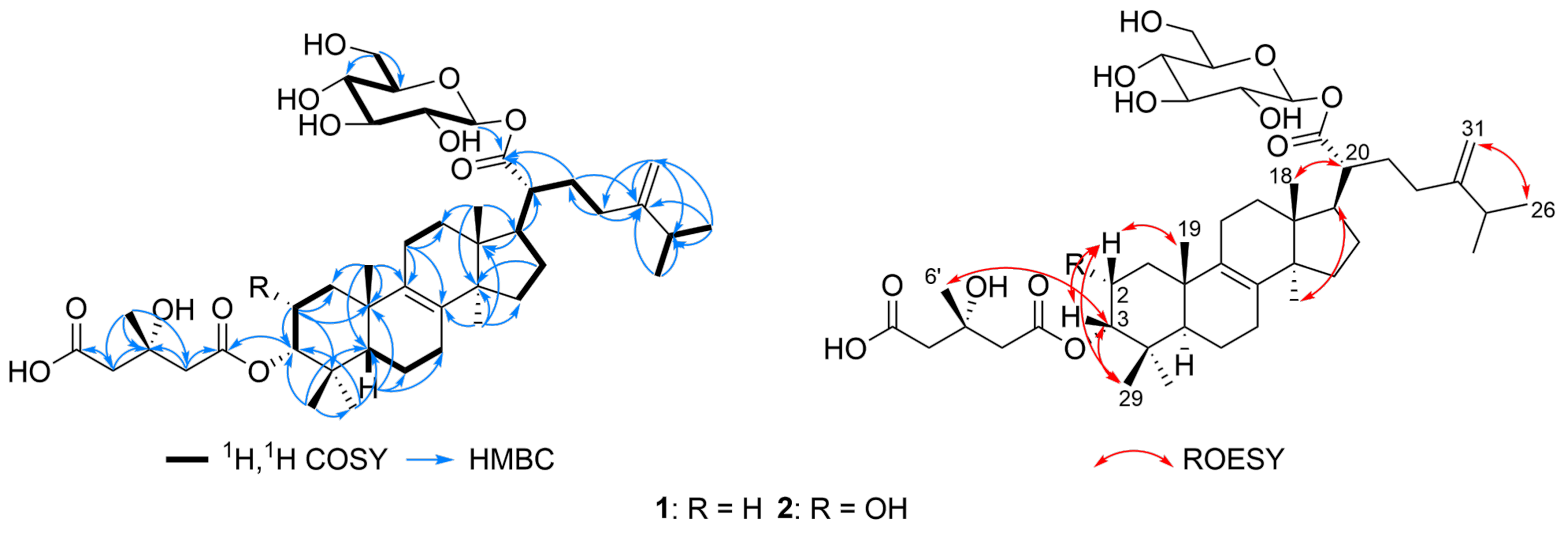
Key ^1^H,^1^H COSY, HMBC, and ROESY correlations of compounds **1** and **2**.

Compound **1** was isolated as an off-white solid powder. Its molecular formula was determined to be C_43_H_68_O_12_ based on HRESIMS results that revealed a sodium adduct ion peak at *m*/*z* 799.4604 ([M + Na]^+^ calcd for C_43_H_68_O_12_Na^+^, 799.4603) indicating the presence of ten degrees of unsaturation in its structure. The ^1^H, ^13^C NMR, and HSQC spectral data of compound **1** (Table 1) revealed the presence of forty-three carbon resonances sorted into eight methyl, fourteen methylenes (one olefinic), ten methine and eleven unprotonated carbon atoms. This includes three carbonyl carbons at δ_C_ 177.4 (C-21), 175.0 (C-5'), and 172.4 (C-1') as well as four olefinic carbon signals at δ_C_ 156.7 (C-24), 136.3 (C-9), 135.2 (C-8), and 107.4 (C-31). The ^1^H and ^13^C NMR spectral data of compound **1** (Table 1) also revealed the presence of a sugar moiety through the presence of the characteristic anomeric proton resonance at δ_H_ 5.49 (d, *J* = 8.1, H-1'') which was directly correlated to the carbon at δ_C_ 95.7 (C-1'') in the HSQC spectrum. The ^1^H,^1^H COSY spectrum of compound **1** (Figure 2) revealed an extended spin system over four aliphatic methines and ending with one aliphatic methylene. By comparing the ^1^H and ^13^C NMR data (Table 1) of the sugar moiety with that reported for a related fungal lanostanoside, ganosinoside A [26] and other glycosidic moieties [27], it was confirmed to be a β-ᴅ-glucopyranosyl residue. The HMBC spectrum of compound **1** (Figure 2) also revealed key correlations from two methylene groups at δ_H_ 2.65/2.69 (H_2_-2') and δ_H_ 2.69/2.72 (H_2_-4') to an ester carbonyl (δ_C_ 172.4, C-1') and a carboxyl (δ_C_ 175.0, C-5'), respectively. In addition, both methylene groups together with a methyl singlet at δ_H_ 1.38 (s, H_3_-6') disclosed key HMBC correlations to a quaternary oxygenated carbon at δ_C_ 70.7 (C-3') suggesting the presence of a 3-hydroxy-3-methylglutaroyl moiety in compound **1** [23].

**Table 1:** ^1^H and ^13^C NMR data of compounds **1** and **2** in methanol-*d*_4_.

Pos.	**1**		**2**	
	δ_C_,^a,b^ type	δ_H_^c^ (multi, *J* (Hz))	δ_C_,^a,b^ type	δ_H_^c^ (multi, *J* (Hz))

1	32.1, CH_2_	1.51 (m, 2H)	40.7, CH_2_	1.55 (t, 12.6, 1H) 1.73 (dd, 12.6, 4.0, 1H)
2	24.2, CH_2_	1.65 (m, overlapped, 1H) 1.92 (m, overlapped, 1H)	66.5, CH	4.02 (ddd, 12.3, 4.3, 2.9, 1H)
3	79.8, CH	4.68 (t, 2.8, 1H)	81.8, CH	4.96 (dd, 2.9, 0.9, 1H)
4	37.8, C		39.0, C	
5	46.8, CH	1.56 (br d, 2.0, 1H)	46.2, CH	1.46 (dd, 13.0, 2.2, 1H)
6	19.1, CH_2_	1.56 (m, overlapped, 1H) 1.66 (m, overlapped, 1H)	18.8, CH_2_	1.55 (m, overlapped, 1H) 1.66 (m, overlapped, 1H)
7	27.1, CH_2_	1.41 (m, overlapped, 2H)	29.8, CH_2_	1.54 (m, overlapped, 1H) 1.64 (m, overlapped, 1H)
8	135.2, C		135.3, C	
9	136.3, C		136.0, C	
10	38.1, C		39.5, C	
11	21.9, CH_2_	2.03 (m, overlapped, 2H)	22.1, CH_2_	2.05 (m, overlapped, 2H)
12	29.9, CH_2_	1.51 (m, overlapped, 1H) 1.61 (m, overlapped, 1H)	29.8, CH_2_	1.53 (m, overlapped, 1H) 1.63 (m, overlapped, 1H)
13	45.6, C		45.6, C	
14	50.7, C		50.6, C	
15	31.5, CH_2_	1.26 (m, overlapped, 1H) 1.67 (m, overlapped, 1H)	31.5, CH_2_	1.27 (m, overlapped, 1H) 1.67 (m, overlapped, 1H)
16	27.1, CH_2_	2.08 (m, overlapped, 2H)	27.0, CH_2_	2.09 (m, overlapped, 2H)
17	48.3, CH	2.14 (m, 1H)	48.3, CH	2.14 (m, overlapped, 1H)
18	16.7, CH_3_	0.81 (s, 3H)	16.6, CH_3_	0.81 (s, 3H)
19	19.5, CH_3_	1.03 (s, 3H)	20.5, CH_3_	1.07 (s, 3H)
20	48.8, CH	2.40 (td, 11.0, 3.5, 1H)	48.9, CH	2.40 (td, 11.0, 3.4, 1H)
21	177.4, C		177.3, C	
22	32.6, CH_2_	1.68 (m, overlapped, 1H) 1.73 (m, overlapped, 1H)	32.6, CH_2_	1.68 (m, overlapped, 1H) 1.73 (m, overlapped, 1H)
23	32.7, CH_2_	1.98 (m, overlapped, 1H) 2.11 (m, overlapped, 1H)	32.7, CH_2_	1.97 (m, overlapped, 1H) 2.12 (m, overlapped, 1H)
24	156.7, C		156.7, C	
25	35.0, CH	2.23 (pd, 6.9, 1.1, 1H)	35.0, CH	2.24 (pd, 6.9, 1.0, 1H)
26	22.3, CH_3_	1.03 (d, 6.8, 3H)	22.3, CH_3_	1.03 (d, 6.9, 3H)
27	22.4, CH_3_	1.01 (d, 6.8, 3H)	22.4, CH_3_	1.01 (d, 6.9, 3H)
28	28.4, CH_3_	0.90 (s, 3H)	28.3, CH_3_	0.92 (s, 3H)
29	22.3, CH_3_	0.95 (s, 3H)	22.1, CH_3_	0.99 (s, 3H)
30	24.7, CH_3_	0.94 (s, 3H)	24.7, CH_3_	0.94 (s, 3H)
31	107.4, CH_2_	4.70 (d, 1.4, 1H) 4.75 (d, 1.4, 1H)	107.4, CH_2_	4.70 (d, 1.4, 1H) 4.75 (d, 1.4, 1H)
1'	172.4, C		172.9, C	
2'	46.5, CH_2_	2.65 (d, 15.1, 1H) 2.69 (d, 15.1, 1H)	46.7, CH_2_	2.71 (d, 14.4, 1H) 2.77 (d, 14.4, 1H)
3'	70.7, C		70.9, C	
4'	45.9, CH_2_	2.69 (d, 14.9, 1H) 2.72 (d, 14.9, 1H)	46.6, CH_2_	2.63 (d, 15.0, 1H) 2.66 (d, 15.0, 1H)
5'	175.0, C		175.3, C	
6'	27.9, CH_3_	1.38 (s, 3H)	27.5, CH_3_	1.39 (s, 3H)
1''	95.7, CH	5.49 (d, 8.1, 1H)	95.7, CH	5.50 (d, 8.3, 1H)
2''	73.9, CH	3.35 (m, overlapped, 1H)	73.9, CH	3.36 (dd, 9.1, 8.2, 1H)
3''	78.4, CH	3.42 (m, overlapped, 1H)	78.4, CH	3.43 (d, 9.1, 1H)
4''	71.2, CH	3.39 (m, overlapped, 1H)	71.2, CH	3.39 (m, overlapped, 1H)
5''	78.7, CH	3.38 (m, overlapped, 1H)	78.7, CH	3.38 (m, overlapped, 1H)
6''	62.6, CH_2_	3.71 (dd, 11.9, 1.8, 1H) 3.81 (dd, 11.8, 4.4, 1H)	62.6, CH_2_	3.71 (dd, 11.8, 4.5, 1H) 3.81 (dd, 11.8, 1.8, 1H)

^a^At 500 MHz; ^b^assigned based on HMBC and HSQC spectra; ^c^at 125 MHz.

These results illustrated that compound **1** is a C31 lanostane-type triterpenoid glucoside. According to the above results, the C31 lanostane-type triterpenoid moiety of compound **1** was identified to be a lanostan-8,24(31)-diene-21-oic acid skeleton supported by 2D NMR cross peaks in the ^1^H,^1^H COSY, HMBC, and HSQC spectra, suggesting a closely related structure to forpinioside A [23,28]. The C-5, C-10, C-13, and C-14 configurations were assigned not only from the biogenetic considerations, but also from careful comparison of the ^1^H and ^13^C NMR data with those of related compounds and ROESY correlations. The orientation of H-3 (δ_H_ 4.68, t, *J* = 2.8 Hz) was determined to be β based on comparing the measured and the reported coupling constants and chemical shifts for related lanostanoid derivatives [13,23,29–30].

Indeed, when the proton H-3 has a β-orientation as in compound **1**, it resonates in the form of a broad singlet or else in the form of a triplet with a small coupling constant indicating its equatorial disposition [31–32]. When this proton has an α-orientation, it resonates as a doublet of doublet with coupling constants around 4.5 and 11 Hz [28,33]. The β-orientation of H-3 in compound **1** was further supported not only by the ROESY correlation observed between H-3 (δ_H_ 4.68, t, *J* = 2.8 Hz) and Me-29 (δ_H_ 0.95, s), but also by the fact that 3-epipachymic acid (**3**) and 3α,25*S*-3-*O*-malonyl-23-oxolanost-8,24(31)-dien-26-oic acid (**4**) were obtained during this investigation from the same extract. Some important ROESY correlations were depicted between Me-30 (δ_H_ 0.94, s) and H-17 (δ_H_ 2.14, m) as well as between H-20 (δ_H_ 2.40, td, *J* = 11.0, 3.5 Hz) and Me-18 (δ_H_ 0.81, s) indicating the α-orientation of H-17 and C-21, respectively. By comparing the HRESIMS, ^1^H and ^13^C NMR data of compound **1** and those reported for forpinioside A [23,28], it was obviously recognized that compound **1** lacks the ester group at δ_H_ 3.62 (OCH_3_-5'; δ_C_ 51.9) on the 3-hydroxy-3-methylglutaroyl substituent. Further confirmation of the positions of the 3-hydroxy-3-methylglutaroyl and β-ᴅ-glucopyranosyl moieties was provided by the HMBC spectrum which exhibited key correlations from H-3 to C-1' and from H-20 (δ_H_ 2.40, td, *J* = 11.0, 3.5 Hz)/H-1'' (δ_H_ 5.49, d, *J* = 8.1 Hz) to a carbonyl carbon at δ_C_ 177.4 (C-21). Hence, their positions were notably confirmed to be at C-3 and C-21, respectively. The ᴅ-configuration of the β-glucopyranosyl unit was assumed to be the one found in related fungal lanostanosides namely; ganosinoside A from *Ganoderma sinense* [26], fomitosides I and J from *Fomitopsis pinicola* [34]. Concerning the configuration at C-3', it was assigned as *S* by careful comparison of the ^1^H and ^13^C NMR data of compound **1** with those of related lanostane derivatives comprising the 3-hydroxy-3-methylglutaryl moiety produced by some fungi including *Fomitopsis* pinicola [23], *Piptoporus betulinus* [13], and *Pholiota populnea* [35]. Furthermore, it is well known that similar biosynthetic pathways might lead to the same product in most of the cases. Based on the aforementioned results, compound **1** was identified as 3α-[(3′*S*)-4′-carboxyl-3′-hydroxy-3′-methylbutanoyloxy]lanosta-8,24(31)-dien-21-oic acid 21-*O*-β-ᴅ-glucopyranoside that was trivially named as forpinioside B.

Compound **2** was obtained as a colourless oil and its molecular formula was determined to be C_43_H_68_O_13_ according to its HRESIMS spectrum that revealed a sodium adduct ion peak at *m*/*z* 815.4550 ([M + Na]^+^ calcd for C_43_H_68_O_13_Na^+^, 815.4552) indicating the presence of an additional oxygen atom compared to compound **1**.

The ^1^H, ^13^C NMR, and HSQC spectral data of compound **2** (Table 1) revealed a close similarity to forpinioside B (**1**) apart from the presence of an additional aliphatic methine proton at δ_H_ 4.02 (ddd, *J* = 12.3, 4.3, 2.9 Hz; δ_C_ 66.5) which exhibited an obvious spin system in the ^1^H,^1^H COSY spectrum (Figure 2) with a methylene group at δ_H_ 1.51/δ_H_ 1.73 (H_2_-1) and a methine proton at δ_H_ 4.96 (H-3). These results suggested that compound **2** features a hydroxy group at C-2 rather than a methylene group as in forpinioside B (**1**). Further confirmation for the suggested position of the hydroxy group at C-2 was provided by the HMBC spectrum (Figure 2) which exhibited clear correlations from H-2 to four carbon resonances at δ_C_ 40.7 (C-1), 81.8 (C-3), 39.0 (C-4), and 39.5 (C-10).

Aside from this difference and by comparing the 1D and 2D NMR spectral data of compounds **1** and **2**, they were closely related derivatives. The relative orientation of H-2 was determined to be β based on its ROESY spectrum (Figure 2) which revealed key NOE correlations to H-3 (δ_H_ 4.96) along with two singlet methyl groups namely, Me-19 (δ_H_ 1.07) and Me-29 (δ_H_ 0.99). Based on the above results, compound **2** was identified as 2α-hydroxy,3α-[(3′*S*)-4′-carboxyl-3′-hydroxy-3′-methylbutanoyloxy]lanosta-8,24(31)-dien-21-oic acid 21-*O*-β-ᴅ-glucopyranoside that was named as forpinioside C.

### Biological activities

Compounds **1**–**4** were tested for their antimicrobial effects against fungi and bacteria; where compound **1** was moderately active against the Gram-positive bacteria *Bacillus subtilis* and *Staphylococcus aureus* at MIC values of 8.3 µg/mL and 16.6 µg/mL, respectively. The antagonism of **1** against *B. subtilis* and *S. aureus* was compared to the positive controls oxytetracycline and gentamycin, with MICs values recorded at 16.6 µg/mL and 0.21 µg/mL, respectively. Compounds **1**, **2**, and **4** exhibited no cytotoxic effects against highly sensitive mammalian cell lines namely; mouse fibroblasts (L929) and human endocervical adenocarcinoma cells (KB3.1), hence no further tests were made on the other cell lines (Table S1 in Supporting Information File 1). However, 3-epipachymic acid (**3**) demonstrated significant cytotoxicity against epidermoid carcinoma cells (A431) (IC_50_ = 5.7 µM) and HeLa cells (KB-3-1) (IC_50_ = 7.0 µM). Moderate cytotoxic effects for compound **3** were also recorded against mouse fibroblasts (L929) (IC_50_ = 15.2 µM), breast cancer cells (MCF-7) (17.6 µM), and prostate cancer cells (PC-3) (18.9 µM).

## Discussion

The introduction of a hydroxy group at C-2 rendered forpinioside C (**2**) inactive in antimicrobial assays compared to forpinioside B (**1**), however; both compounds were not active in the cytotoxicity assay. Recent structure–activity relationship (SAR) studies have indicated a key role played by the hydroxy group at C-3 in cytotoxic effects of lanostane triterpenoids [23,36]. According to a study by Wang et al. (2023) [36], synthetic derivatives of pachymic acid demonstrated moderate to high potency against cancer cells in the presence of a 3-OH group. Notably, hydrolysis of the C3-acetoxy group in pachymic acid to tumulosic acid increased the activity of the compound compared to the positive control (cisplatin), in some instances [36]. Concomitantly, the oxidation of the hydroxy group at C-3 into a ketone moiety diminished the activity [31]. Similar findings on the related polyporenic acid B, with significant IC_50_ values between 8.4–12.2 µM [22], confirms the probable crucial role played by the 3-OH group in improving the potency. The presence of the 3-*O*-methylglutaroyl functionality in compound **1** seemed to be responsible for its antibiotic effects. The introduction of a 2-OH group in compound **2** could have interfered with its 3-*O*-methylglutaroyl group, rendering it inactive in the antimicrobial assay. However, as previous hinted by studies on similar derivatives [22], the cytotoxic effects of compound **1** and related analogues could be realized by C-3 hydrolysis.

In 2010, Liu et al. also demonstrated the major role played by changes at C-16 on antibacterial effects of *F. pinicola* steroids [33]. The acetylation at C-16 reduced the potency compared to the corresponding congener with a hydroxy group [33]. In our case, the C-16 hydroxy group seemed to play a major role in the cytotoxicity instead, as indicated by the active compound 3-epipachymic acid (**3**). Intriguingly, other compounds lacking the hydroxy group at C-16 were inactive, suggesting the probable role played by a 16-OH in inducing cytotoxicity. In addition, another study demonstrated that a lanostanoid glycoside derivative with a glucosyl ester at the C-21 carboxylic acid group was active in a cytotoxic activity assay, whereas the galactosyl ester counterpart was inactive [36]. Similarly, the carboxylic acid group at C-21 was shown to be a key player in lanostane triterpenoid cytotoxicity; increased activity was demonstrated either by its esterification with glucose or by its reduction into a hydroxymethylene group unlike its presence as a free carboxylic acid moiety [23]. Thus, our findings provide further insights into the SARs of lanostanoid triterpenoids and expands the database of their bioactive compounds for subsequent studies.

## Conclusion

The genus *Fomitopsis* remains to be a prolific source of several metabolites with health-promoting effects. We provide a new evidence of lanostanoid glycosides **1** and **2** from solid-state cultivated cultures of *F. carnea*, with forpinioside B (**1**) proving to be a potential antimicrobial. In addition, our findings provide significant insights to decipher the SARs of the lanostanoid triterpenoids.

## Experimental

### General experimental methods

The samples were analyzed on an amaZon speed ETD ion trap mass spectrometer (Bruker Daltonics, Bremen, Germany) for HPLC-DAD/MS in positive and negative ionization modes. The HPLC (Dionex UltiMate 3000 UHPLC, Thermo Fisher Scientific Inc., Waltham, MA, USA) system’s stationary phase was composed of a C18 Acquity UPLC BEH column (Waters, Milford, MA, USA). The solvent system was as follows; deionized H_2_O + 0.1% formic acid (FA, v/v) (solvent A) and acetonitrile (ACN) + 0.1% FA (v/v) (solvent B). The separation gradient was operated as follows; 5% B for 0.5 min, 5% B to 100% B for 20 min, and 100% B for 10 min. The flow rate was maintained at 0.6 mL/min and the UV–vis detection made at 210 nm and 190–600 nm.

The isolates were analyzed on a MaXis ESI-TOF (time-of-flight) mass spectrometer (Bruker Daltonics) for the HRESIMS data, in the positive ionization mode. This was coupled to an Agilent 1260 series HPLC–UV system (Agilent Technologies, Santa Clara, CA, USA). The HPLC system’s stationary phase was composed of a C18 Acquity UPLC BEH column (Waters). The mobile phase employed was as follows; deionized H_2_O + 0.1% FA (v/v) (solvent A) and ACN + 0.1% FA (v/v) (solvent B). The separation gradient was operated as follows; 5% B for 0.5 min, 5% B to 100% B within 19.5 min and at 100% B for 5 min. The flow rate was maintained at 0.6 mL/min and UV–vis detection made at 200–600 nm. The calculation of the compounds’ molecular formulas was performed using the Smart Formula algorithm of the Compass DataAnalysis software (Bruker Daltonics, version 4.4 SR1).

The NMR spectra were recorded on an Avance III 700 (Bruker Biopsin, ^1^H: 700 MHz, ^13^C: 176 MHz) and/or an Avance III 500 (Bruker Biospin, Ettlingen, Germany, ^1^H: 500 MHz, ^13^C: 125 MHz) instruments. Deuterated methanol and dimethyl sulfoxide were used in the measurements. Coupling constants were reported in hertz (Hz) and chemical shifts in parts per million (ppm). The reference values 2.49 ppm and 3.31 ppm were used for the residual proton signals in the calibration of ^1^H NMR spectra for CD_3_SOCD_3_ and CD_3_OD, respectively. Likewise, the reference values of 39.5 and 49.1 for the deuterated solvents CD_3_SOCD_3_ and CD_3_OD, respectively, were used in ^13^C NMR spectra calibration. An Anton Paar MCP-150 Polarimeter (Graz, Austria), was used to measure the optical rotations on a 100 mm path length and sodium D line at 589 nm. The sample concentration was 1.0 mg/mL in MeOH. A Shimadzu UV–vis 2450 spectrophotometer (Kyoto, Japan) was used to measure the UV–vis spectra at a concentration of 0.02 mg/mL in MeOH.

The chemicals and solvents (analytical and HPLC grade) were purchased from Merck KGaA (Darmstadt, Germany), AppliChem GmbH (Darmstadt, Germany), Carl Roth GmbH & Co. KG (Karlsruhe, Germany), and Avantor Performance Materials (Deventer, Netherlands). An in-house Purelab^®^ flex water purification system (Veolia Water Technologies, Celle, Germany), was used in the preparation of deionized water.

### Fungal material examined

The current study specimen was collected by one of the authors C.D in Kakamega National Park, Kenya (0°17'3.19" N 34°45'8.24" E) from a dead tree trunk. A voucher specimen is deposited at the Mycothèque de la Université catholique de Louvain (MUCL), Belgium under designated number MUCL 56078. The fungal specimen was identified morphologically by author C.D by comparison of its morphological traits to close relatives.

### Fermentation and metabolites extraction

Mycelial cultures of *F. carnea* were fermented on solid and liquid-state media based on previously established methodologies [37]. Essentially, the preparation of solid-state media was as follows: 90 mg of rice was weighed out into 10 × 500 mL Erlenmeyer flasks, each containing 90 mL of deionized H_2_O and autoclaved. In addition, 20 × 200 mL Erlenmeyer flasks containing cotton seed flour (Q6/2) liquid-state fermentation media were prepared as follows: media components (ᴅ-glucose 2.5 g/L, glycerol 10 g/L, cotton seed flour 5 g/L in distilled water), were mixed, pH adjusted to 7.2, and autoclaved. The media flasks were inoculated with 10 mycelial plugs (5 mm) each and incubated for 28 days, under shaking and static conditions for liquid and solid cultures, respectively. The cultures were extracted after incubation following the aforementioned established procedures to afford 0.5 g (mycelial) and 1.5 g (supernatant) ethyl acetate extracts for Q6/2 cultures and 3.44 g ethyl acetate extract for rice cultures.

### Isolation of compounds **1–4**

Purification of the rice and Q6/2 media cultures was achieved by using a reversed-phase HPLC system (PLC 2020; Gilson, Middleton, WI, USA), equipped with a VP Nucleodur 100-5 C-18 ec packed column (25 × 40 mm, 7 μm, Macherey-Nagel) as stationary phase. The liquid phase consisted of solvent A and solvent B. The elution gradients for the Q6/2 and rice extracts were identical and were performed as follows: isocratic conditions at 5% solvent B for 10 min, followed by an increase to 20% in 3 min, 20% to 90% within 50 min, 90% to 100% in 5 min and finally isocratic conditions of solvent B at 100% for 30 min. The flow rate in each case was 40 mL/min and UV detections were carried out at 210, 254, and 350 nm. The mycelial Q6/2 extract yielded compound **3** (6.4 mg, *t*_R_ = 86–88 min) and the rice extract yielded compounds **4** (3.4 mg, *t*_R_ = 53 min), **1** (13.3 mg, *t*_R_ = 57–58 min), and **2** (4.4 mg, *t*_R_ = 51 min).

Forpinioside B (**1**): off-white solid powder; 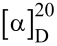 +9 (*c* 0.1, MeOH); UV–vis (MeOH) λ_max_, nm (log ε): 196.5 (1.7); NMR data (^1^H NMR: 500 MHz, ^13^C NMR: 125 MHz in methanol-*d*_4_) see Table 1; HRMS–ESI (*m*/*z*): [M + Na]^+^ calcd for C_43_H_68_NaO_12_^+^, 799.4603; found, 799.4604; [2M + Na]^+^ calcd for C_86_H_136_NaO_13_^+^, 1575.9314; found, 1575.9321.

Forpinioside C (**2**): pale yellow oil; 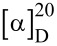 +30 (*c* 0.1, MeOH); UV–vis (MeOH) λ_max_, nm (log ε): 196.5 (1.7); NMR data (^1^H NMR: 500 MHz, ^13^C NMR: 125 MHz in methanol-*d*_4_) see Table 1; HRMS–ESI (*m*/*z*): [M – H_2_O + H]^+^ calcd for C_43_H_67_O_12_^+^, 775.4627; found, 775.4625; [M + Na]^+^ calcd for C_43_H_68_NaO_13_^+^, 815.4552; found, 815.4550; [2M + H]^+^ calcd for C_86_H_137_O_26_^+^, 1607.9212; found, 1607.9210.

### Antimicrobial assays

A wide array of microbial test pathogens were used for the antimicrobial assays according to our previously laid-out protocol [38]. The minimum inhibitory concentrations (MICs) of the compounds were determined via serial dilutions on 96-well microtiter plates. In brief, 20 µL aliquots of the compounds (1 mg/mL), in methanol were pipetted into the 96-well plates consisting of the respective test microorganisms, with a concentration range between 67 µg/mL and 0.5 µg/mL.

### Cytotoxicity assays

MTT (3-(4,5-dimethylthiazol-2-yl)-2,5-diphenyltetrazolium bromide) test was used in determining the cytotoxicity (IC_50_) of the isolated compounds as previously established [38–39]. The mammalian cell lines (mouse fibroblasts L929, adenocarcinomic human alveolar basal epithelial cells A549, HeLa cells KB-3-1, breast cancer cells MCF-7, epidermoid carcinoma cells (A431), and prostate cancer cells PC-3) were obtained from the DSMZ collection (Braunschweig, Germany).

## Supporting Information

File 1 HRESIMS profiles and NMR spectroscopic data of **1**, **2** and **4** in CD_3_OD, and of **3** in (CD_3_)_2_S=O; half inhibitory concentrations (IC_50_) for various mammalian cell lines as well as minimum inhibitory concentrations (MIC) of **1**–**4** for bacteria, yeasts and filamentous fungi.
