# Supplementary material for: Two new lanostanoid glycosides isolated from a Kenyan polypore Fomitopsis carnea
Source: Beilstein J Org Chem. 2023 Aug 2;19:1161–9. doi: 10.3762/bjoc.19.84 (PMC10407780; doi:10.3762/bjoc.19.84)
Supplement: File 1 — HRESIMS profiles and NMR spectroscopic data of 1, 2 and 4 in CD3OD, and of 3 in (CD3)2S=O; half inhibitory concentrations (IC50) for various mammalian cell lines as well as minimum inhibitory concentrations (MIC) of 1–4 for bacteria, yeasts and filamentous fungi. [file Beilstein_J_Org_Chem-19-1161-s001.pdf]

BEILSTEIN JOURNAL OF ORGANIC CHEMISTRY

Supporting Information

for

Two new lanostanoid glycosides isolated from a Kenyan polypore *Fomitopsis carnea*

Winnie Chemutai Sum, Sherif S. Ebada, Didsanutda Gonkhom, Cony Decock, Rémy Bertrand Teponno, Josphat Clement Matasyoh and Marc Stadler

Beilstein J. Org. Chem. **2023,** *19,* 1161–1169. doi:10.3762/bjoc.19.84

HRESIMS profiles and NMR spectroscopic data of 1, 2 and 4 in CD_3OD, and of 3 in $(CD_3)_2S{=}O$; half inhibitory concentrations (IC_{50}) for various mammalian cell lines as well as minimum inhibitory concentrations (MIC) of 1–4 for bacteria, yeasts and filamentous fungi

License and Terms: This is a supporting information file under the terms of the Creative Commons Attribution License (https://creativecommons.org/licenses/by/4.0). Please note that the reuse, redistribution and reproduction in particular requires that the author(s) and source are credited and that individual graphics may be subject to special legal provisions.
The license is subject to the *Beilstein Journal of Organic Chemistry* terms and conditions: (https://www.beilstein-journals.org/bjoc/terms)

**Table of contents**	Page
Table S1. Cytotoxicity (IC_{50}) and antimicrobial activity (MIC) of **1**–**4**.	S2
Figure S1. LRESIMS spectra of **1**.	S3
Figure S2. HRESIMS spectra of **1**.	S4
Figure S3. ^{1}H NMR spectrum of **1** in methanol-d_4 at 500 MHz	S5
Figure S4. ^{13}C NMR spectrum of **1** in methanol-d_4 at 125 MHz	S6
Figure S5. ^{1}H,^{1}H COSY spectrum of **1** in methanol-d_4 at 500 MHz.	S7
Figure S6. HMBC spectrum of **1** in methanol-d_4 at 500 MHz.	S8
Figure S7. HSQC spectrum of **1** in methanol-d_4 at 500 MHz.	S9
Figure S8. ROESY spectrum of **1** in methanol-d_4 at 500 MHz.	S10
Figure S9. LRESIMS spectra of **2**.	S11
Figure S10. HRESIMS spectra of **2**.	S12
Figure S11. ^{1}H NMR spectrum of **2** in methanol-d_4 at 700 MHz.	S13
Figure S12. ^{13}C NMR spectrum of **2** in methanol-d_4 at 175 MHz.	S14
Figure S13. ^{1}H,^{1}H COSY spectrum of **2** in methanol-d_4 at 700 MHz.	S15
Figure S14. HMBC spectrum of **2** in methanol-d_4 at 700 MHz.	S16
Figure S15. HSQC spectrum of **2** in methanol-d_4 at 700 MHz.	S17
Figure S16. ROESY spectrum of **2** in methanol-d_4 at 700 MHz.	S18
Figure S17. LRESIMS spectra of **3**.	S19
Figure S18. HRESIMS spectra of **3**.	S20
Figure S19. ^{1}H NMR spectrum of **3** in DMSO-d_6 at 500 MHz.	S21
Figure S20. ^{13}C NMR spectrum of **3** in DMSO-d_6 at 125 MHz.	S22
Figure S21. ^{1}H,^{1}H COSY spectrum of **3** in DMSO-d_6 at 500 MHz.	S23
Figure S22. HMBC spectrum of **3** in DMSO-d_6 at 500 MHz.	S24
Figure S23. HSQC spectrum of **3** in DMSO-d_6 at 500 MHz.	S25
Figure S24. ROESY spectrum of **3** in DMSO-d_6 at 500 MHz.	S26
Figure S25. LRESIMS spectra of **4**.	S27
Figure S26. HRESIMS spectra of **4**.	S28
Figure S27. ^{1}H NMR spectrum of **4** in methanol-d_4 at 700 MHz.	S29
Figure S28. ^{13}C NMR spectrum of **4** in methanol-d_4 at 175 MHz.	S30
Figure S29. ^{1}H,^{1}H COSY spectrum of **4** in methanol-d_4 at 700 MHz.	S31
Figure S30. HMBC spectrum of **4** in methanol-d_4 at 700 MHz.	S32
Figure S31. HSQC spectrum of **4** in methanol-d_4 at 700 MHz.	S33
Figure S32. ROESY spectrum of **4** in methanol-d_4 at 700 MHz.	S34

Table S1. Cytotoxicity (IC_{50}) and antimicrobial activity (MIC) of **1**–**4**.

	IC_{50} (µM)				Positive Control
**Test Cell Line**	**1**	**2**	**3**	**4**	**Epothilone B (nM)**
Mouse fibroblast (L929)	n.a.	n.a.	**15.2**	n.a.	0.65
Human endocervival adenocarcinoma (KB3.1)	n.a.	n.a.	**7.0**	n.a.	0.17
Human prostate carcinoma (PC-3)	n.d.	n.d.	**18.9**	n.d.	0.09
Human breast adenocarcinoma (MCF-7)	n.d.	n.d.	**17.6**	n.d.	0.07
Human ovarian cancer (SKOV-3)	n.d.	n.d.	**47.3**	n.d.	0.09
Human epidermoid carcinoma (A431)	n.d.	n.d.	**5.7**	n.d.	0.06
Human lung carcinoma (A549)	n.d.	n.d.	**47.3**	n.d.	0.05
**Test Microorganism**	**MIC (µg/mL)**				**Positive Control (µg/mL)**
Staphylococcus aureus	**16.6**	n.i.	n.i.	n.i.	0.21^{G}
Escherichia coli	n.i.	n.i.	n.i.	n.i.	0.42^{G}
Bacillus subtilis	**8.3**	n.i.	n.i.	n.i.	16.6^{O}
Pseudomonas aeruginosa	n.i.	n.i.	n.i.	n.i.	0.21^{G}
Pichia anomala	n.i.	n.i.	n.i.	n.i.	16.6^{N}
Candida albicans	n.i.	n.i.	n.i.	n.i.	8.3^{N}
Acinetobacter baumanii	n.i.	n.i.	n.i.	n.i.	0.52^{C}
Chromobacterium violaceum	n.i.	n.i.	n.i.	n.i.	1.70^{G}
Schizosaccharomyces pombe	n.i.	n.i.	n.i.	n.i.	8.30^{N}
Mucor hiemails	n.i.	n.i.	n.i.	n.i.	8.30^{N}
Rhodotorula glutinis	n.i.	n.i.	n.i.	n.i.	4.20^{N}
Mycobacterium smegmatis	n.i.	n.i.	n.i.	n.i.	1.70^{K}

n.a.: No activity. n.i.: No inhibition up to 67 µg/mL. n.d.: Not determined.

G: Gentamycin; O: Oxytetracycline; N: Nystatin; C: Ciprofloxacin; K: Kanymycin.

Generic Display Report

**Analysis Info**		Acquisition Date	22.07.2022 16:39:44
Analysis Name	E:\Volume D\HZI Data\Winnie\3_Fomitopsis carnea\Amazon-20230417T160801Z-001\Amazon\Abund rice Run1 F14_GC6_01_40653.d		
Method	40653.m	Operator	esu
Sample Name	Abund rice Run1 F14	Instrument	amaZon speed
Comment			

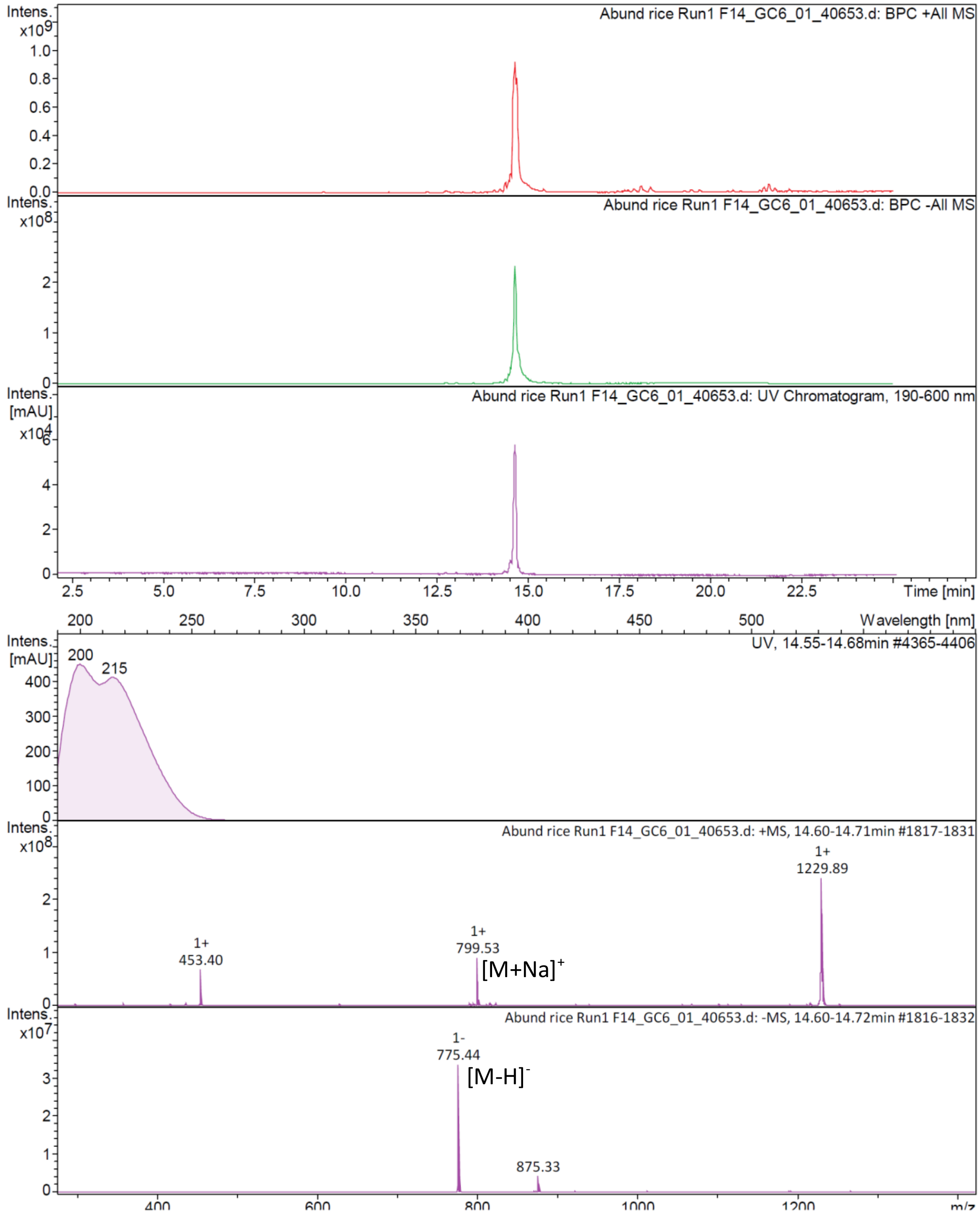

Bruker Compass DataAnalysis 4.4 printed: 18.04.2023 17:51:22 by: mk-mwisnmr Page 1 of 1

Figure S1. LRESIMS of **1**.

Generic Display Report

Analysis Info

Analysis Name	E:\Volume D\HZI Data\Winnie\3_Fomitopsis carnea\Maxis\20230417T160759Z-001\Maxis\Abundisporus_496R1F14_17_01_11010.d	Acquisition Date	15.11.2022 13:22:30
Method	[illegible]	Operator	[illegible]
Sample Name	Abundisporus_496 R1F14	Instrument	maXis
Comment	Screening01<br>Waters Acquity UPLC BEH C_18 1,7um 2.1x50mm		

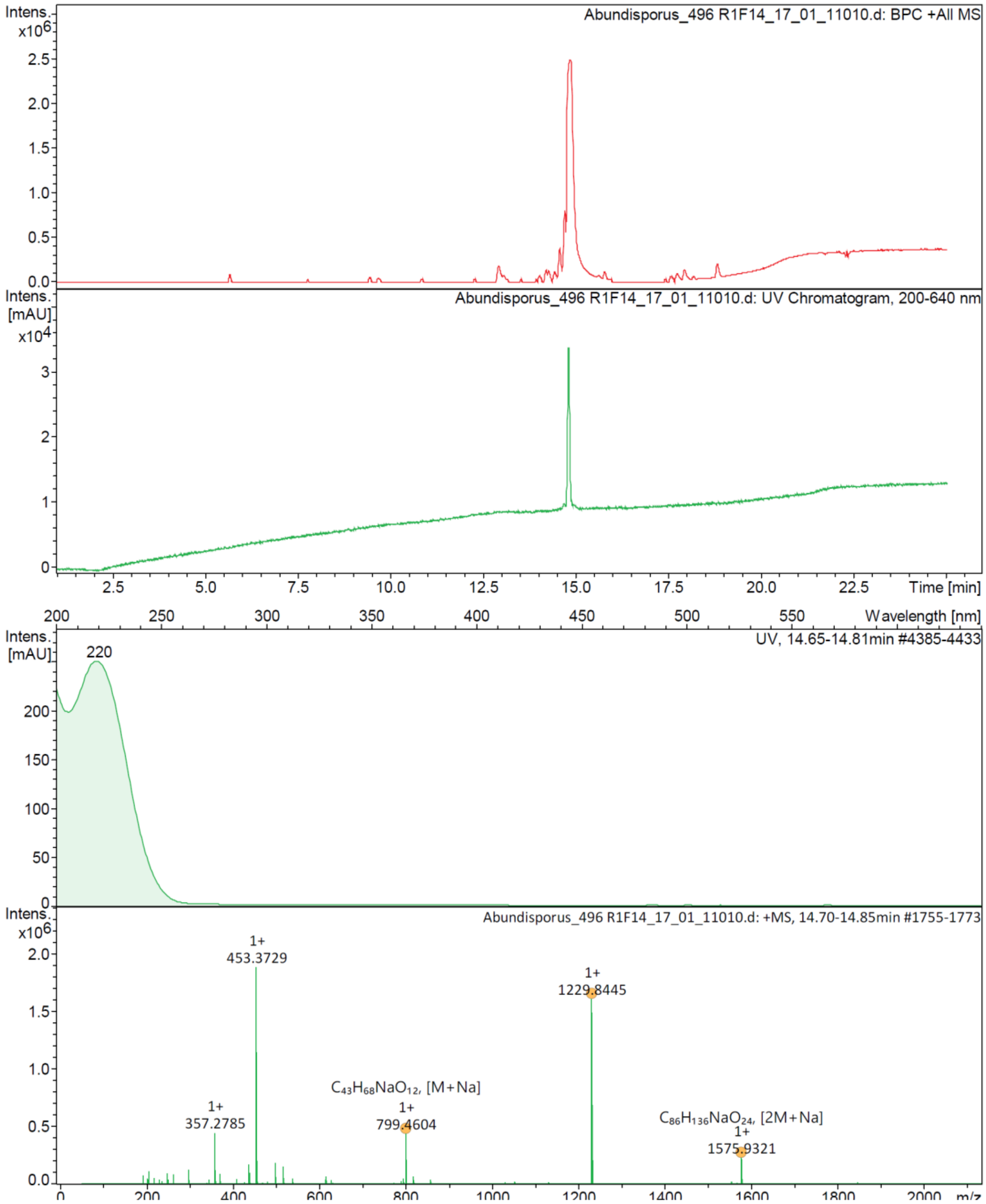

Figure S2. HRESIMS of **1**.

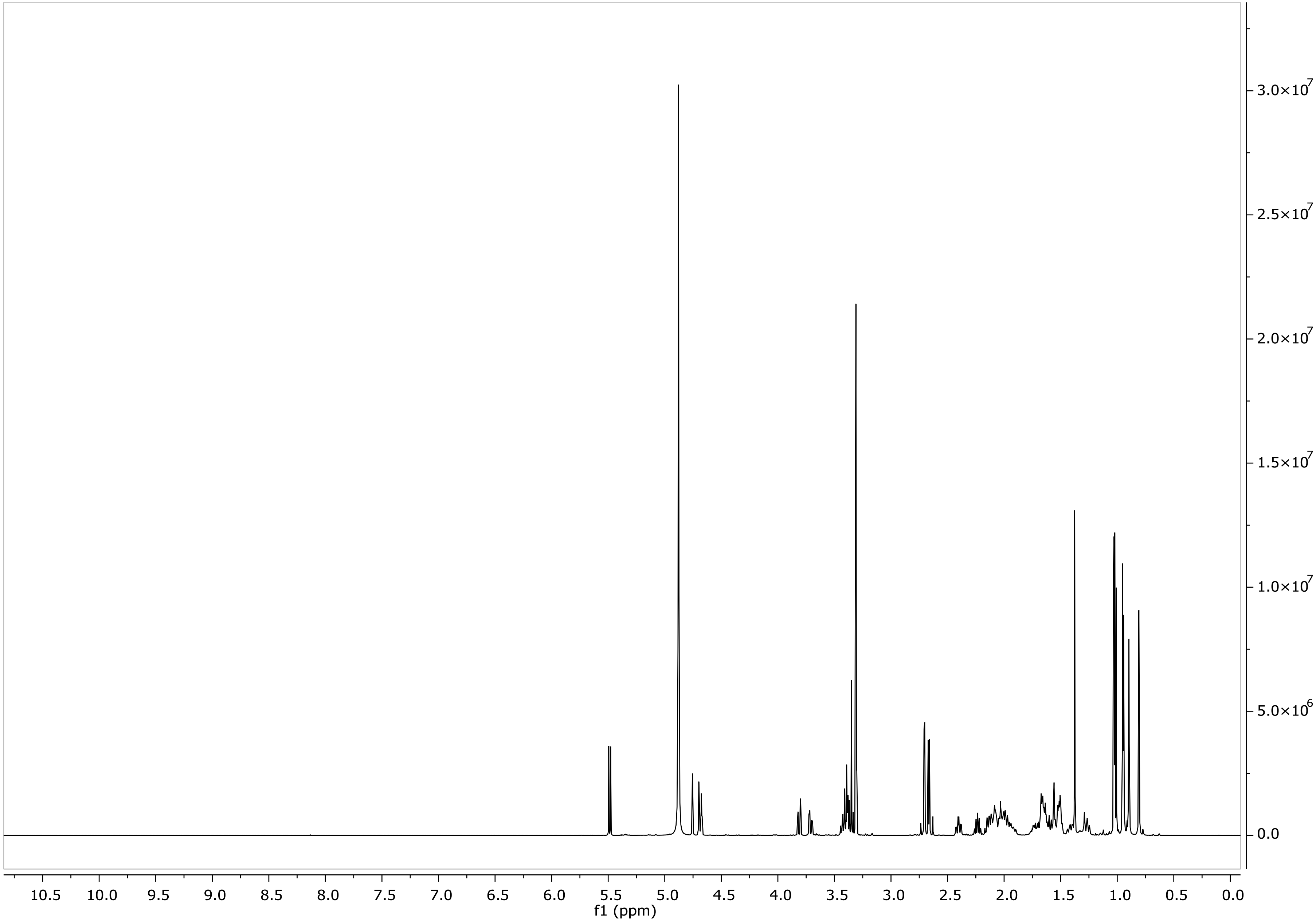

Figure S3. ^{1}H NMR spectrum of **1** in methanol-d_4 at 500 MHz.

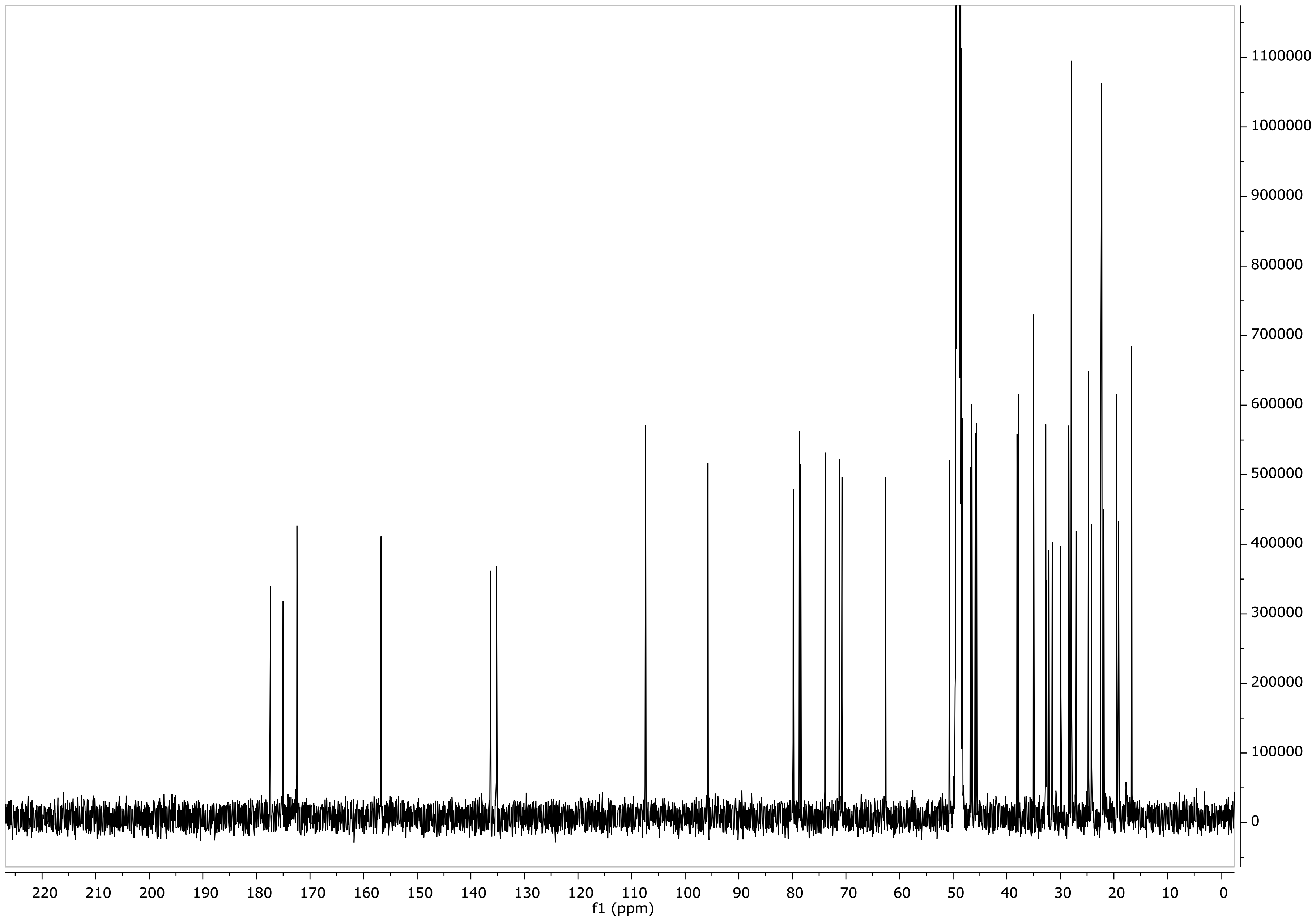

Figure S4. ^{13}C NMR spectrum of **1** in methanol-d_4 at 125 MHz.

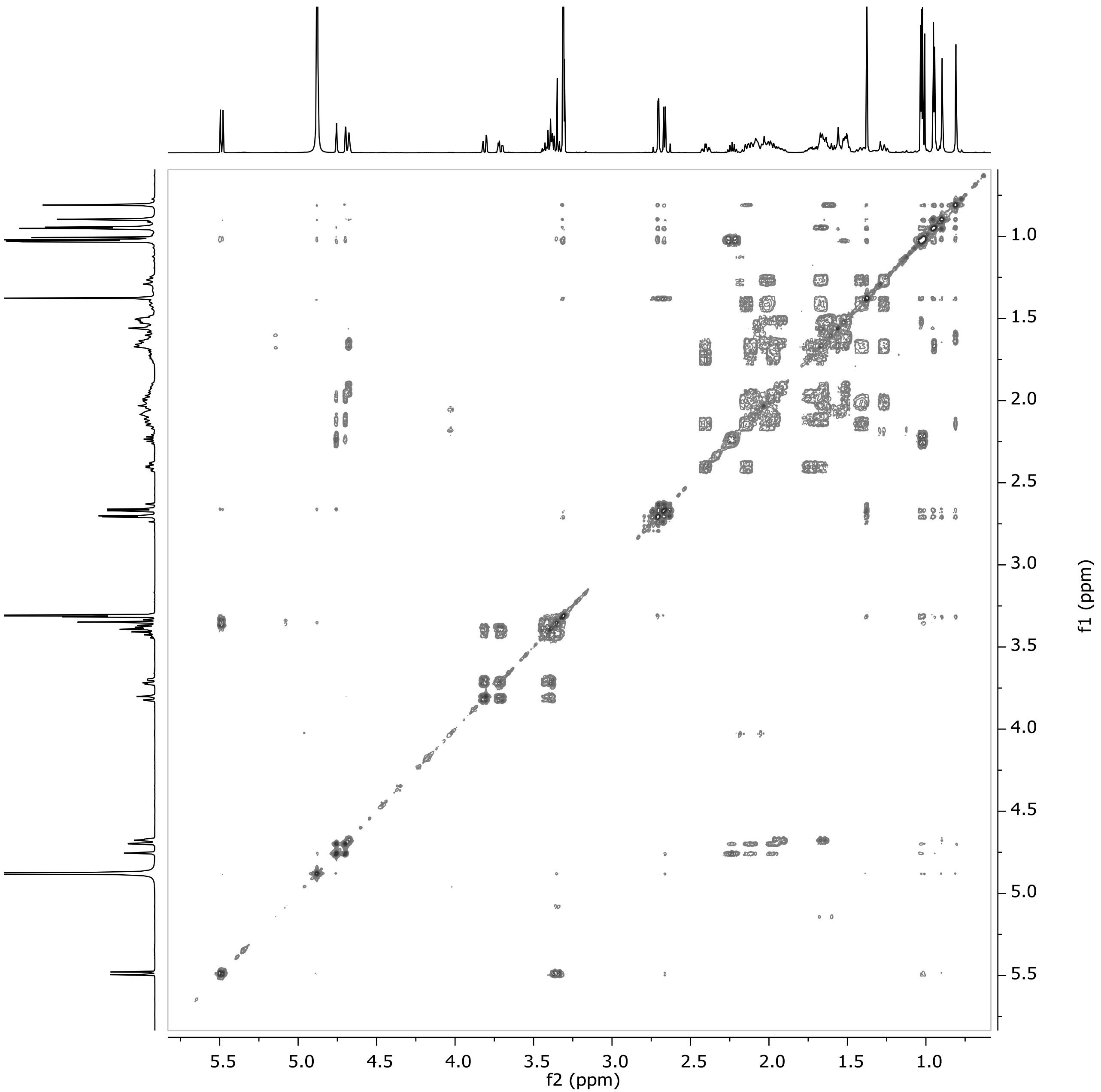

Figure S5. ^{1}H,^{1}H COSY spectrum of **1** in methanol-d_4 at 500 MHz.

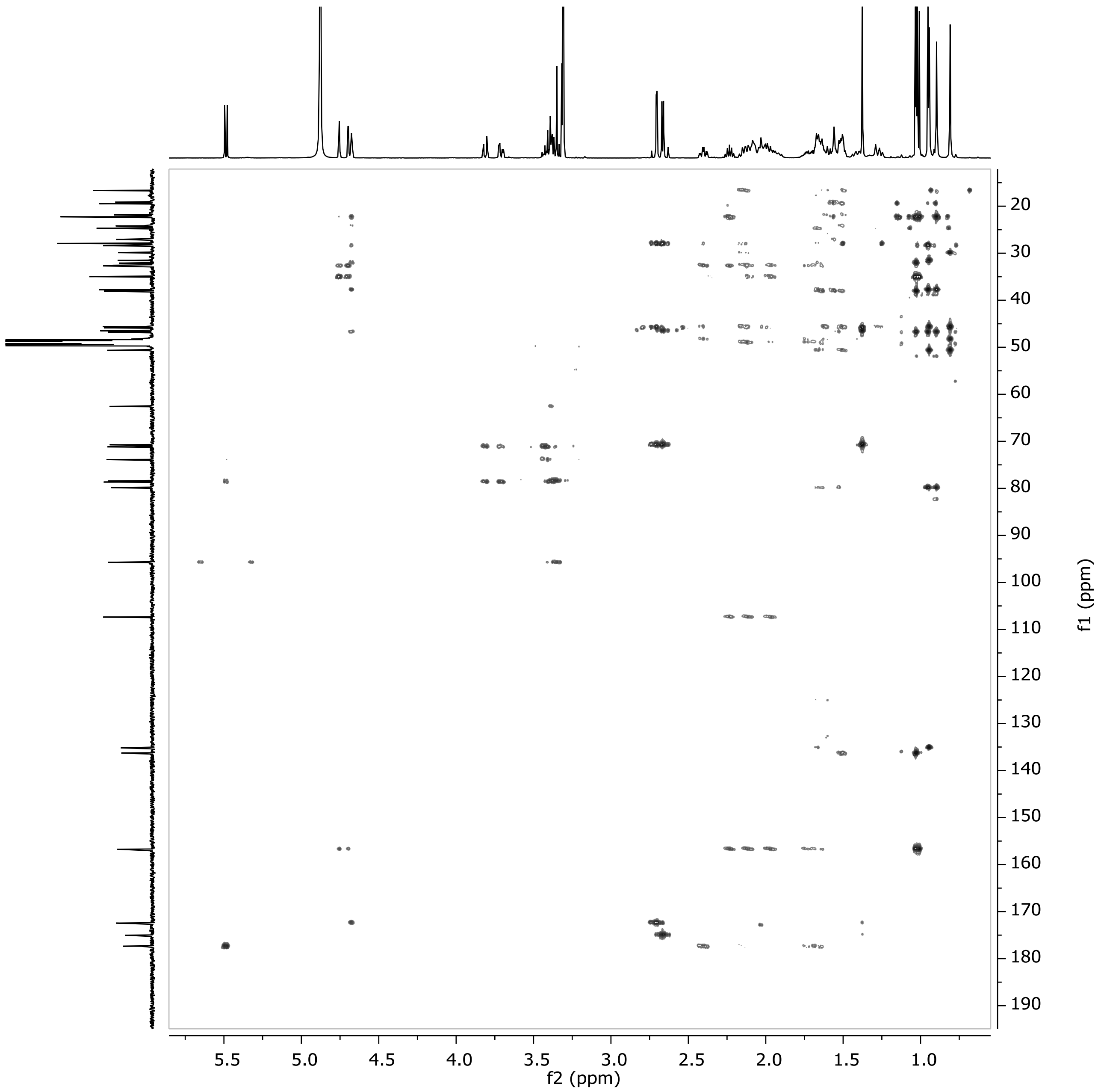

Figure S6. HMBC spectrum of **1** in methanol-d_4 at 500 MHz.

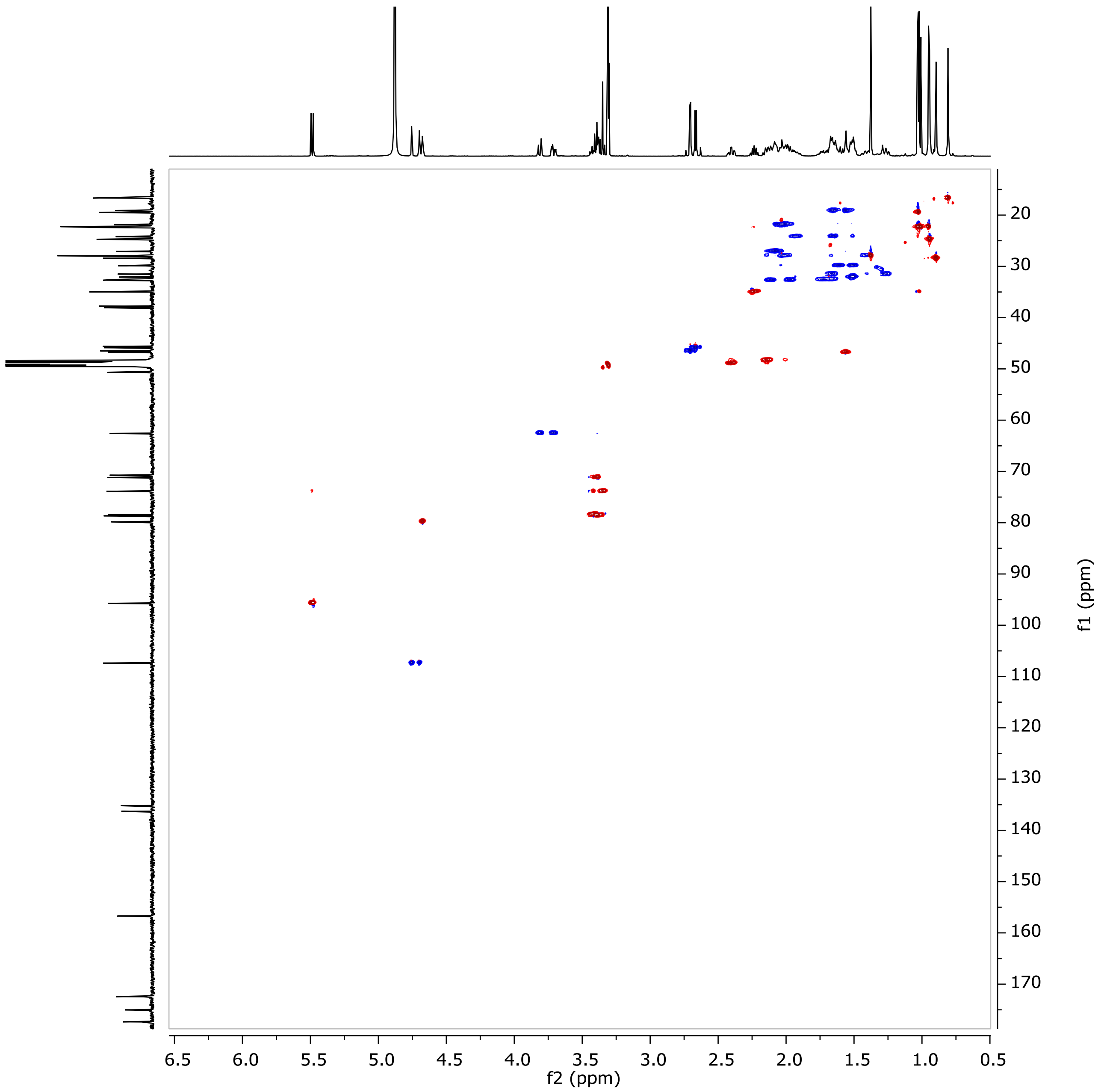

Figure S7. HSQC spectrum of **1** in methanol-d_4 at 500 MHz.

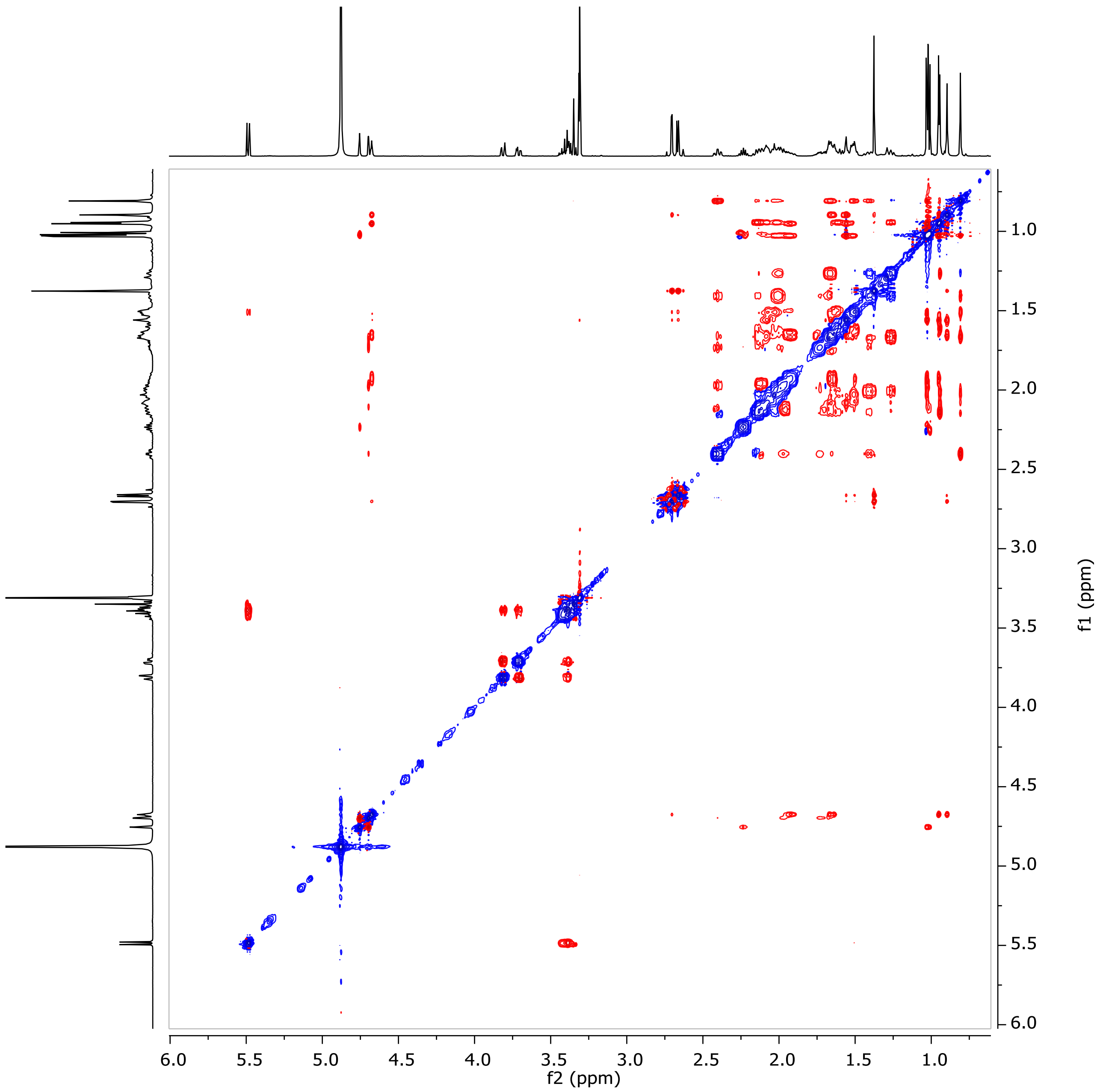

Figure S8. ROESY spectrum of **1** in methanol-d_4 at 500 MHz.

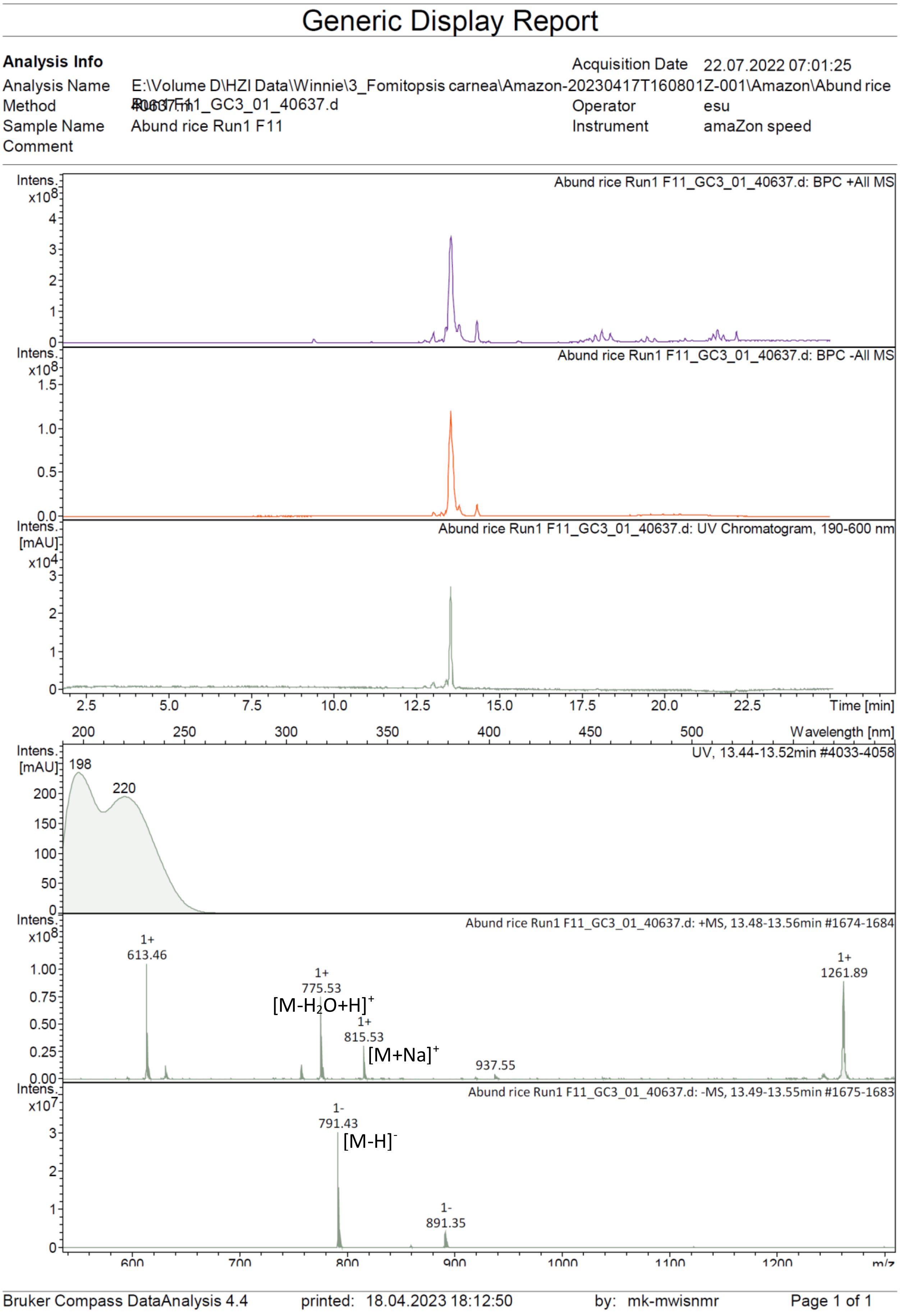

Figure S9. LRESIMS of **2**.

Generic Display Report

Analysis Info

Analysis Name F:\Volume D\HZI Data\Winnie\3_Fomitopsis pomea\Maxis\20230417T160759Z-001\Maxis\Abundisporus_494 R1F11_15_01_11008.d

Method pos_saline_15000_screening_ms_100_2500_line.m

Sample Name Abundisporus_494 R1F11

Comment Screening01

Waters Acquity UPLC BEH C_18 1,7um 2.1x50mm

Acquisition Date 15.11.2022 12:20:30

Operator [illegible]

Instrument maXis

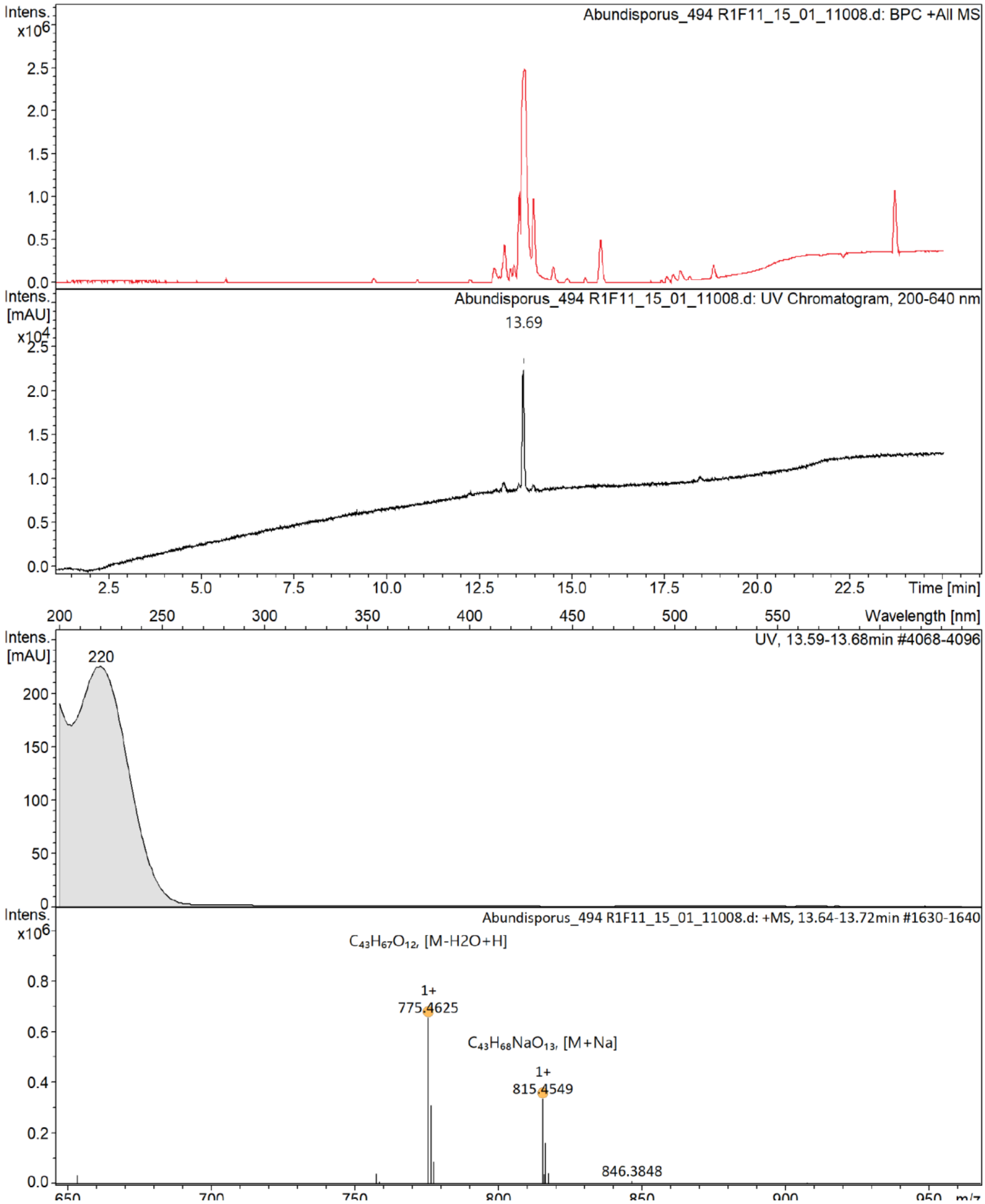

Bruker Compass DataAnalysis 4.4 printed: 11.05.2023 11:59:19 by: sel22 Page 1 of 1

Figure S10. HRESIMS of **2**.

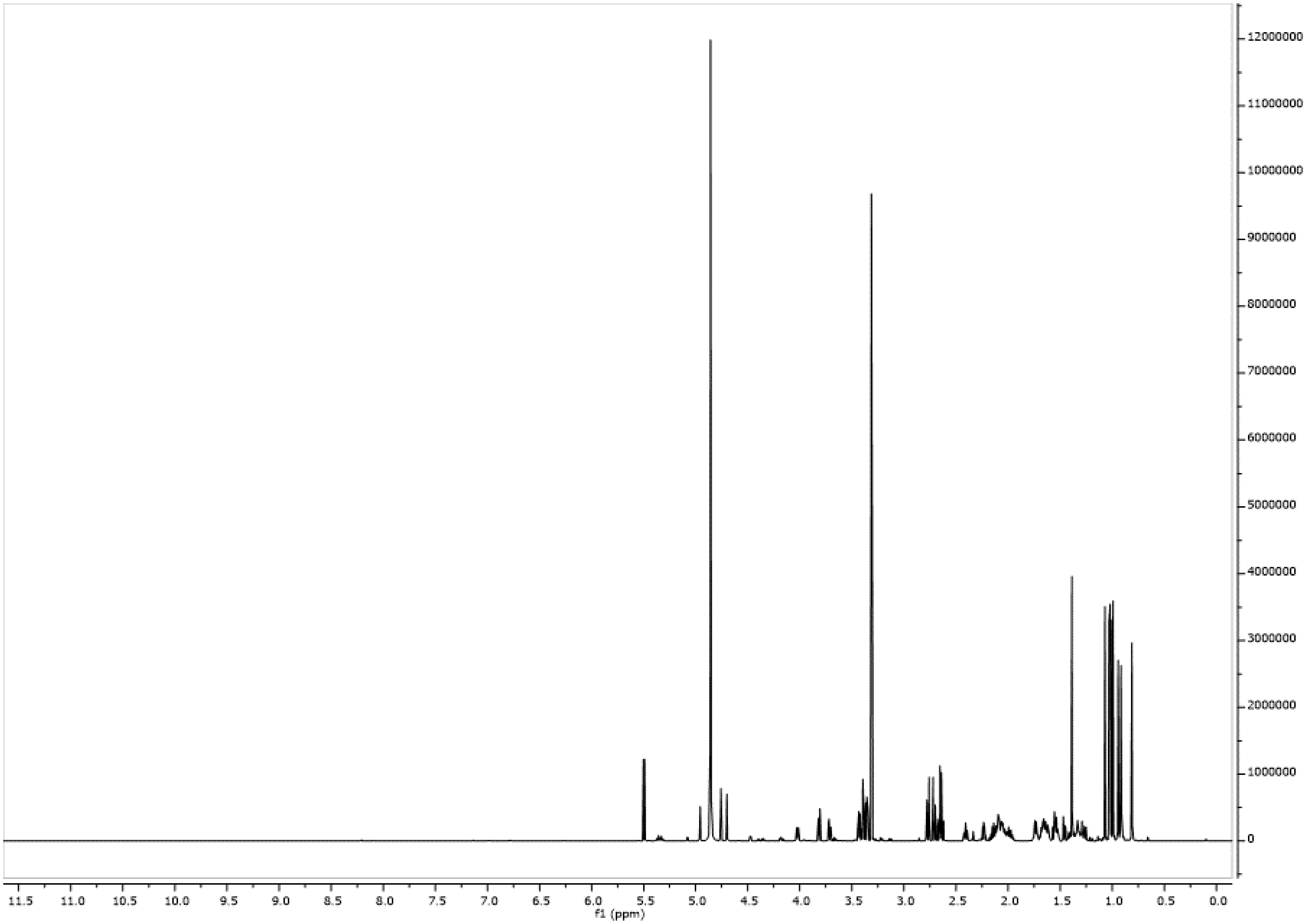

Figure S11. ^{1}H NMR spectrum of **2** in methanol-d_4 at 700 MHz.

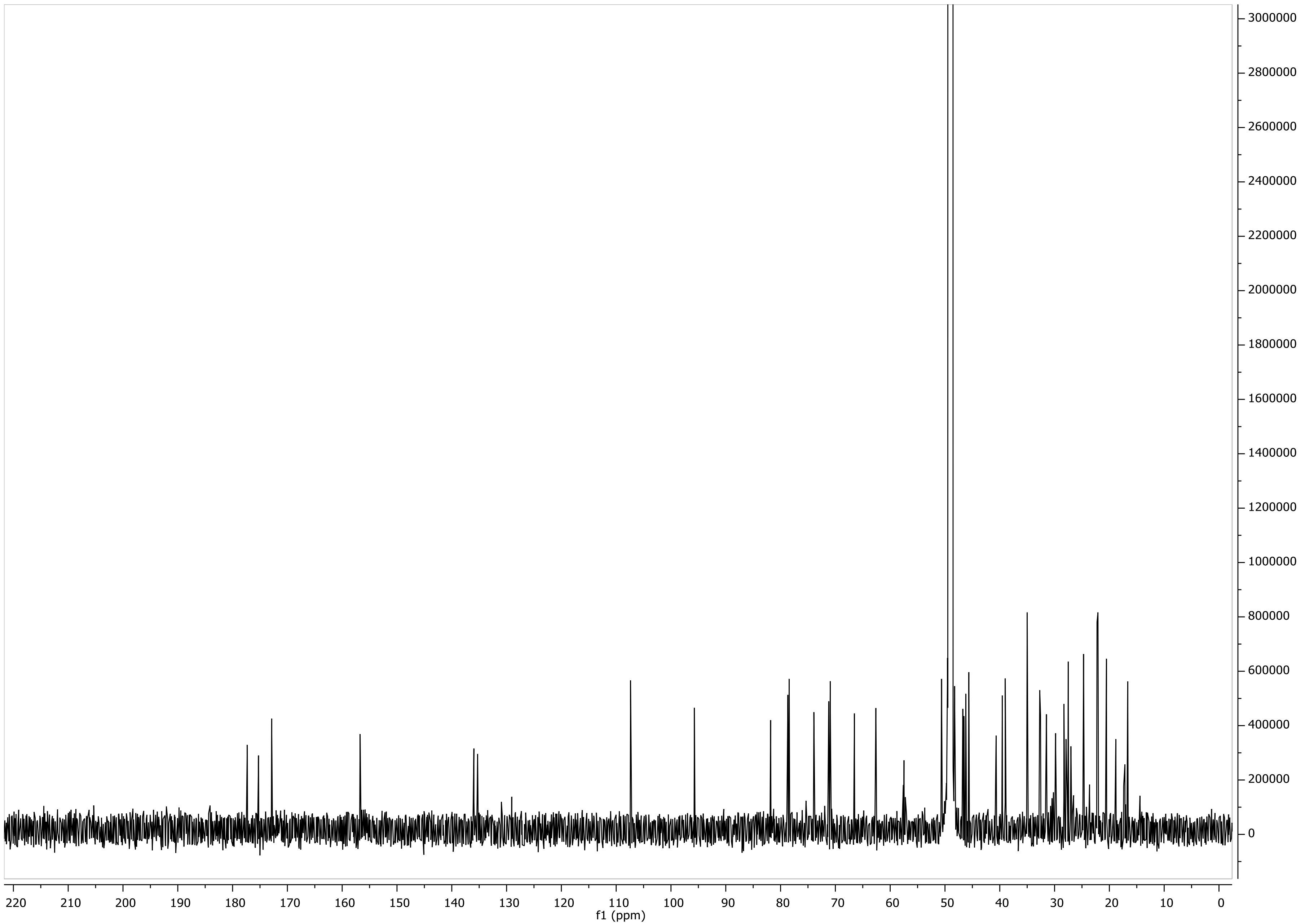

Figure S12. ^{13}C NMR spectrum of **2** in methanol-d_4 at 175 MHz.

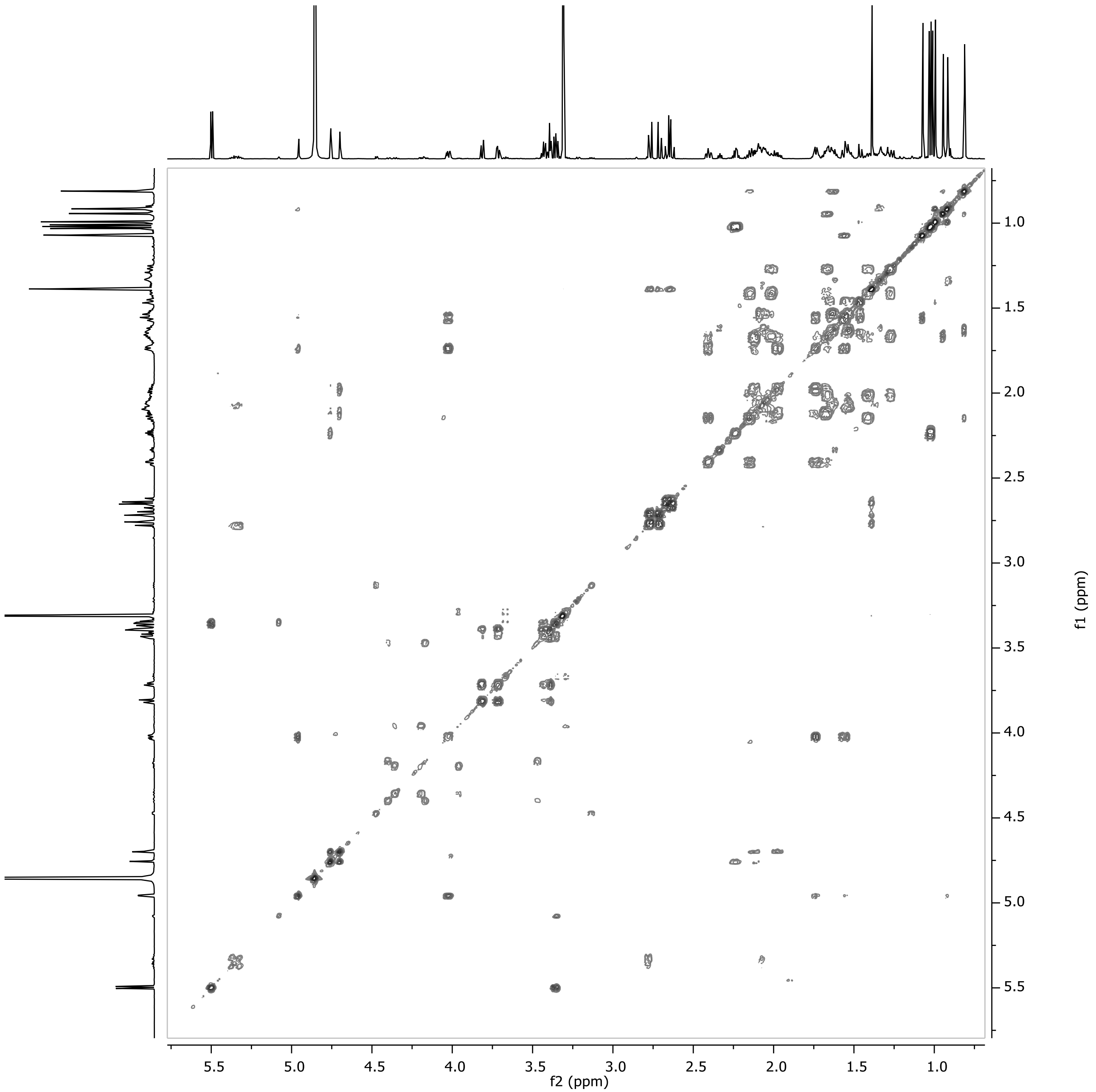

Figure S13. $^{1}H,^{1}H$ COSY spectrum of **2** in methanol-d_4 at 700 MHz.

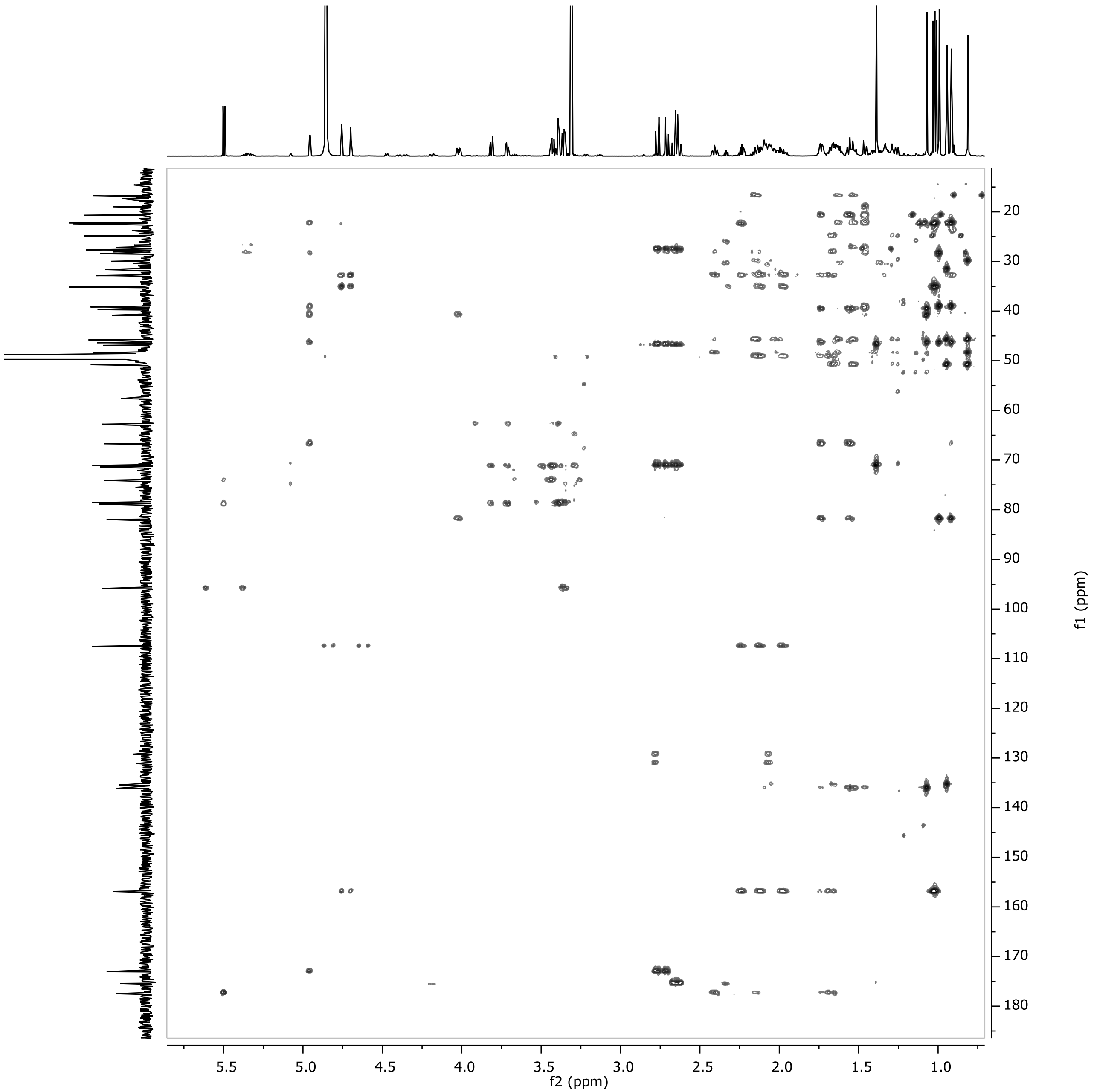

Figure S14. HMBC spectrum of **2** in methanol-d_4 at 700 MHz.

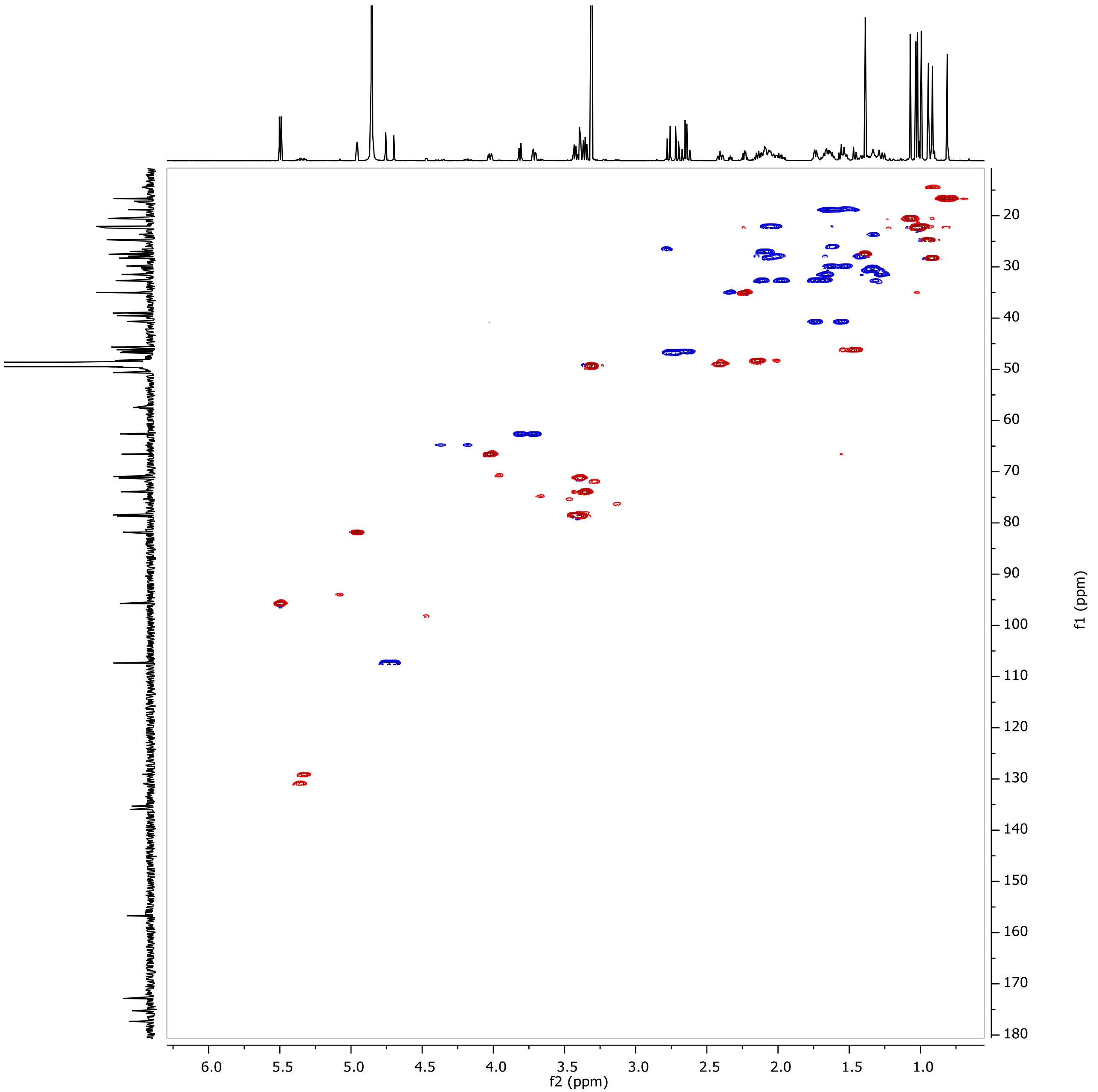

Figure S15. HSQC spectrum of **2** in methanol-d_4 at 700 MHz.

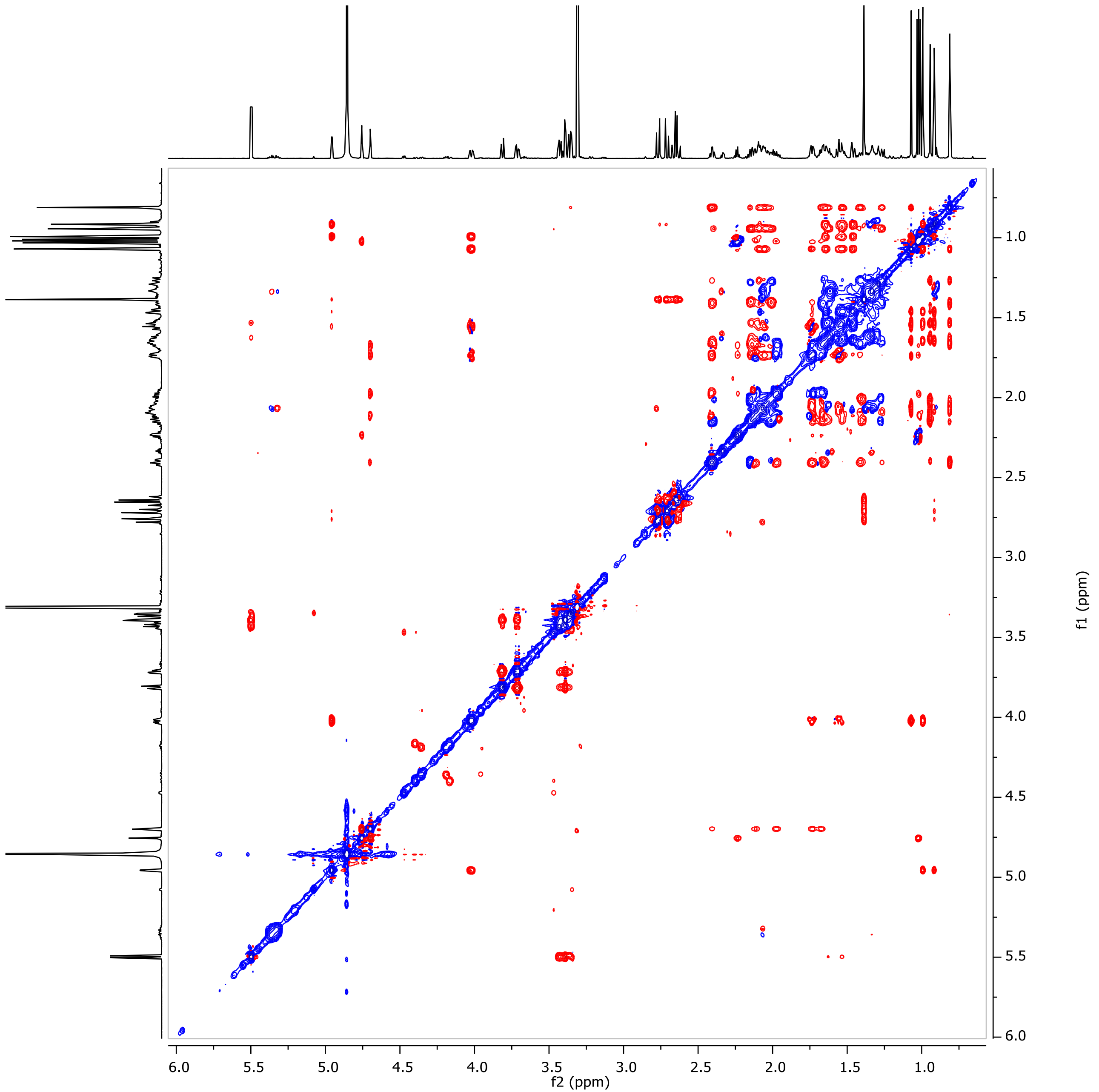

Figure S16. ROESY spectrum of **2** in methanol-d_4 at 700 MHz.

Generic Display Report

Analysis Info

Analysis Name	E:\Volume D\HZI Data\Winnie\3_Fomitopsis 39693.am\Amazon-20230417T160801Z-001\Amazon\Abund_Mycelium_F18_GD2_01_39693.d	Acquisition Date	10.06.2022 05:18:49
Method		Operator	esu
Sample Name	Abund_Mycelium_F18	Instrument	amaZon speed
Comment			

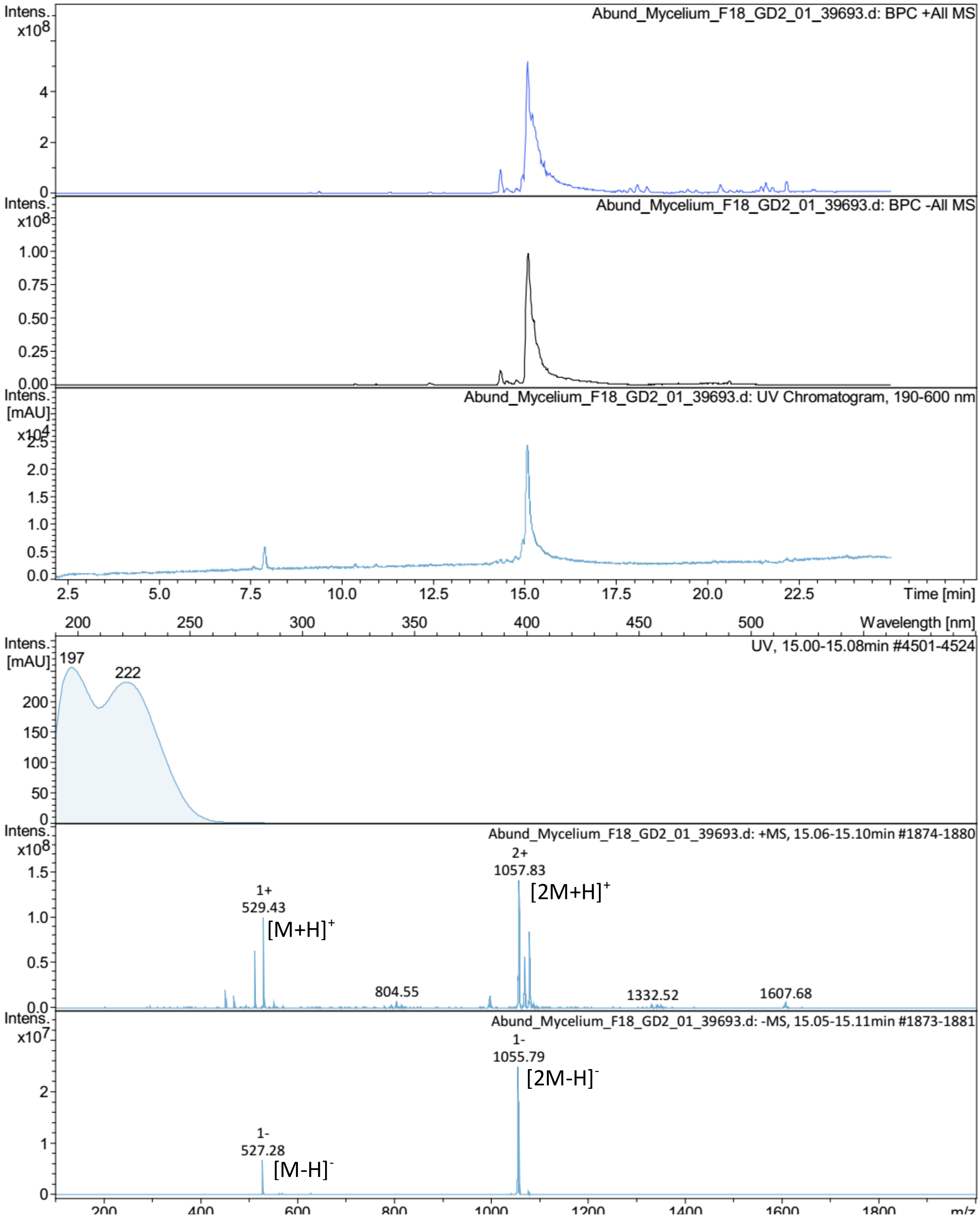

Bruker Compass DataAnalysis 4.4 printed: 18.04.2023 17:52:46 by: mk-mwisnmr Page 1 of 1

Figure S17. LRESIMS of **3**.

Generic Display Report

Analysis Info

Analysis Name	E:\Volume D\HZI Data\Winnie\3_Fomitopsis [illegible]\Abundisporus_258 F18 DMSO_13_01_11006.d	Acquisition Date	15.11.2022 11:18:31
Method	[illegible]	Operator	[illegible]
Sample Name	Abundisporus_258 F18 DMSO	Instrument	maXis
Comment	Screening01<br>Waters Acquity UPLC BEH C_18 1,7um 2.1x50mm		

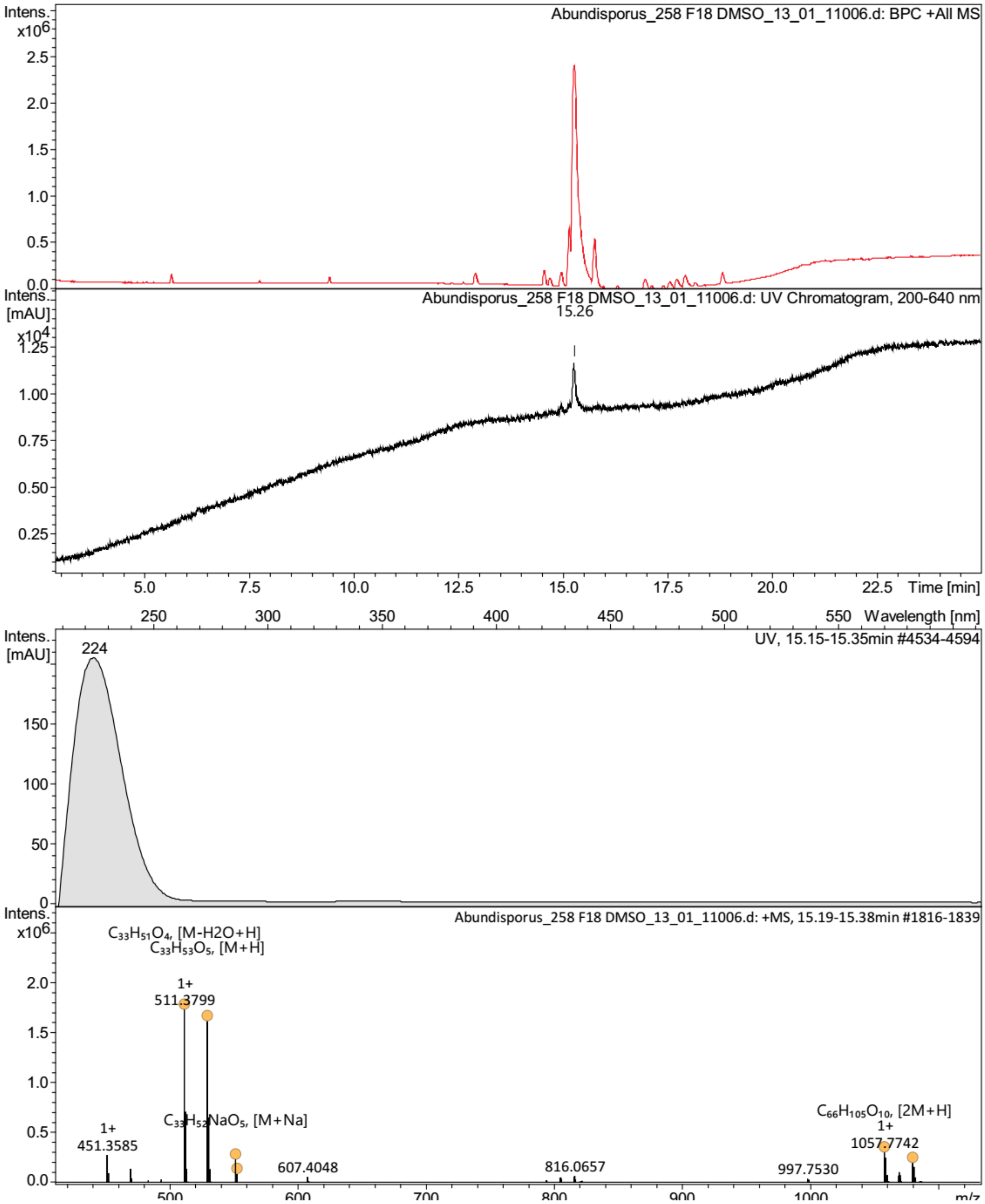

Bruker Compass DataAnalysis 4.4 printed: 18.04.2023 18:07:13 by: mk-mwisnmr Page 1 of 1

Figure S18. HRESIMS of **3**.

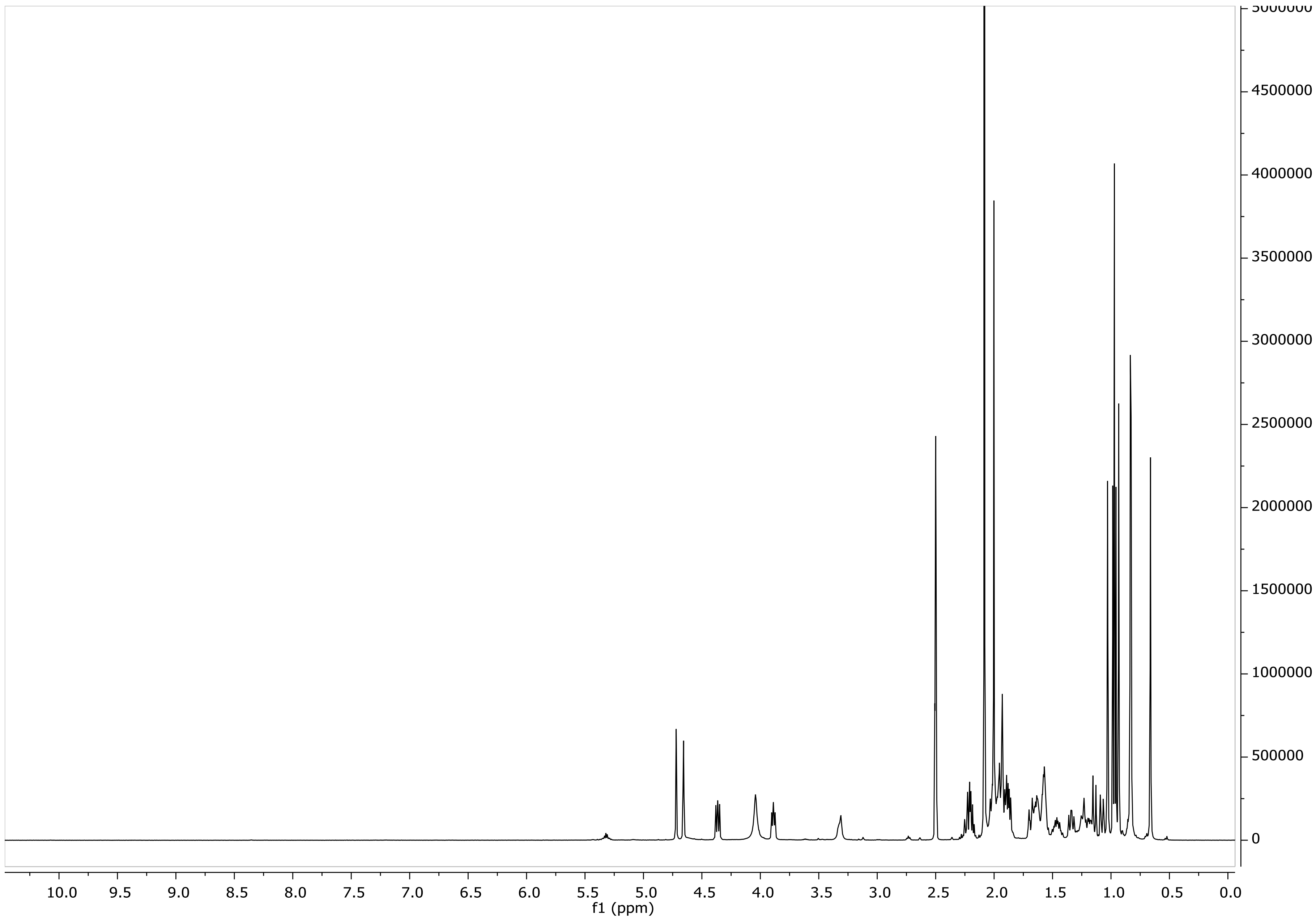

Figure S19. ^{1}H NMR spectrum of **3** in DMSO-d_6 at 500 MHz.

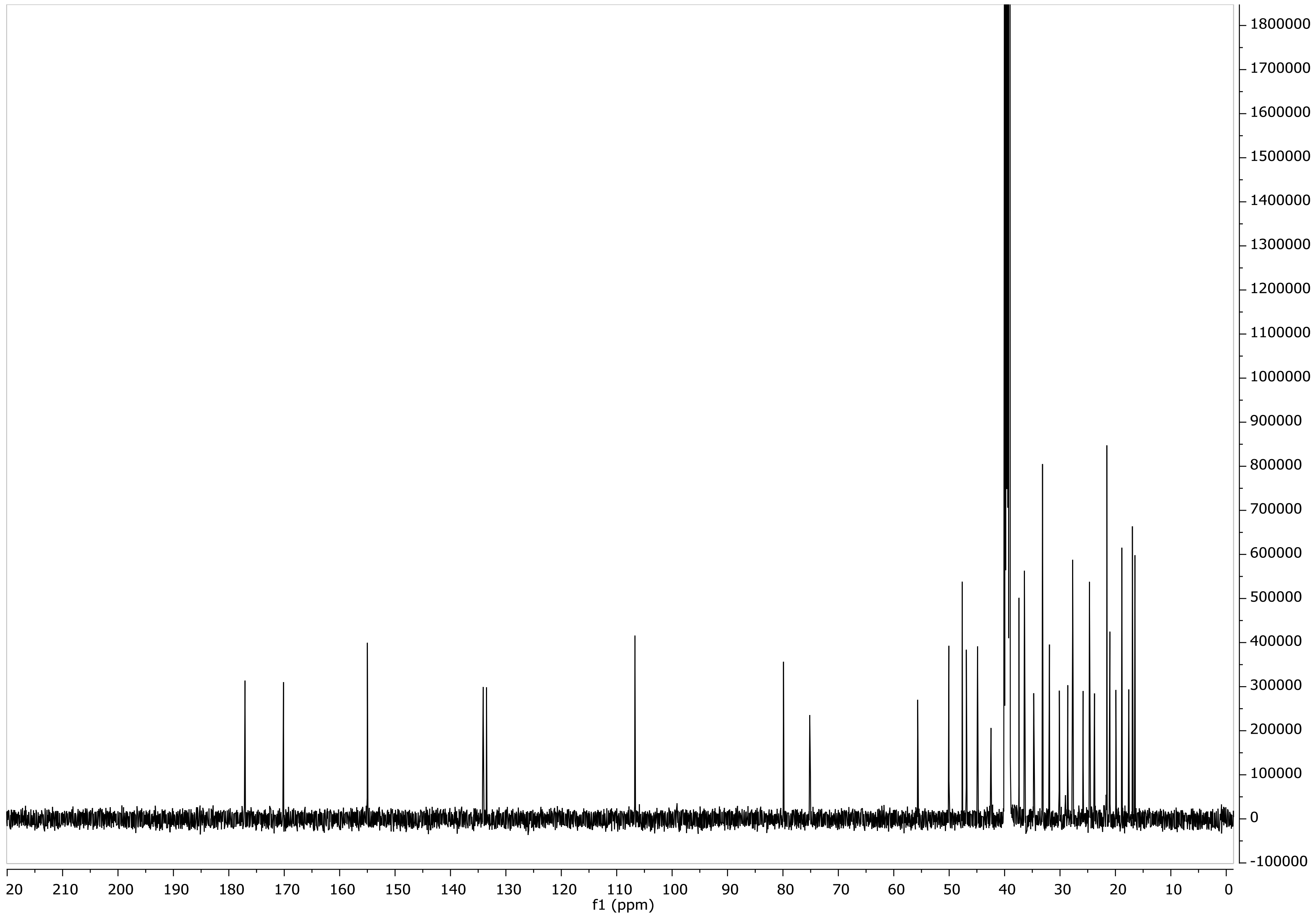

Figure S20. ^{13}C NMR spectrum of **3** in DMSO-d_6 at 125 MHz.

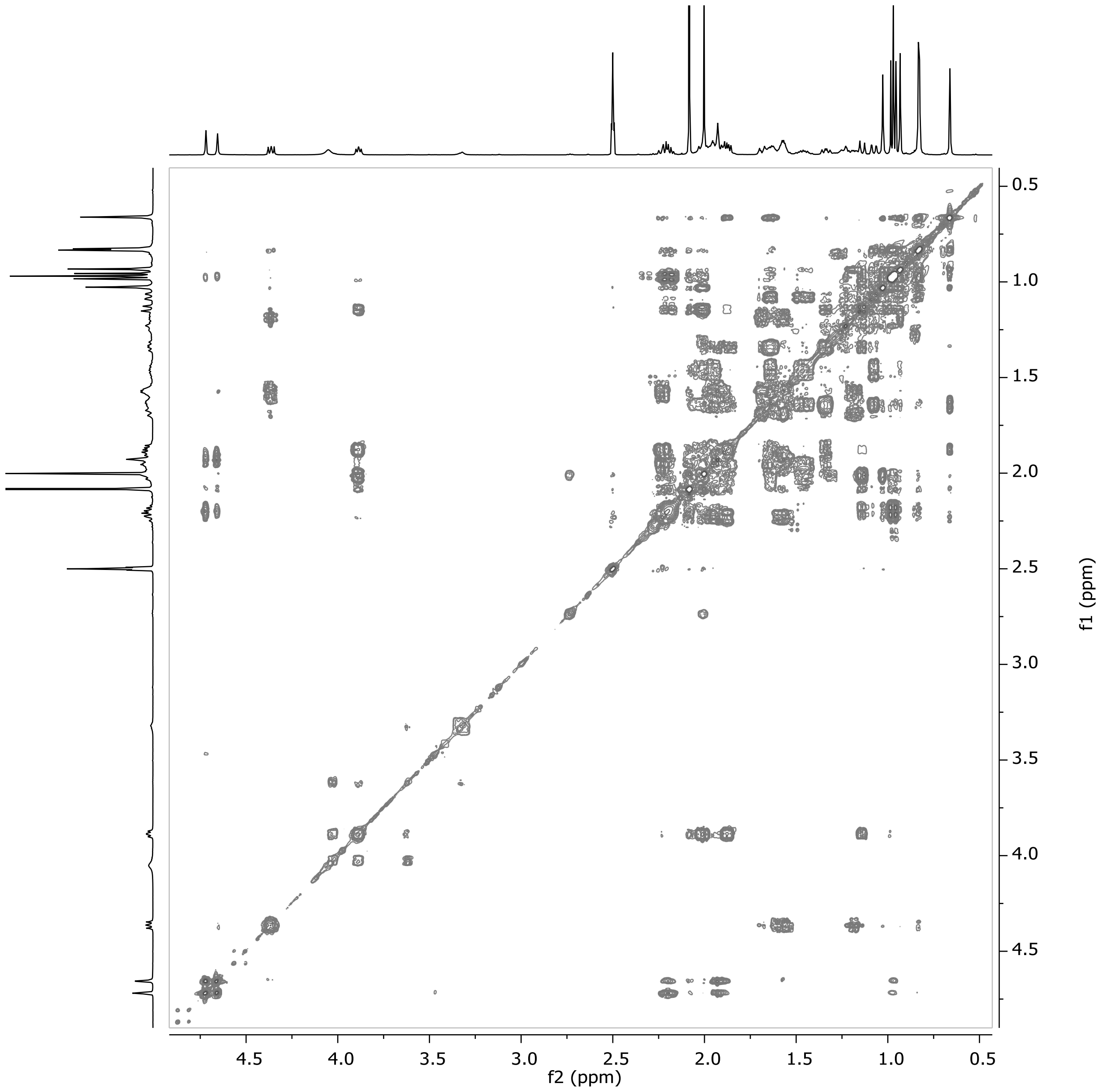

Figure S21. $^{1}H,^{1}H$ COSY spectrum of **3** in DMSO-d_6 at 500 MHz.

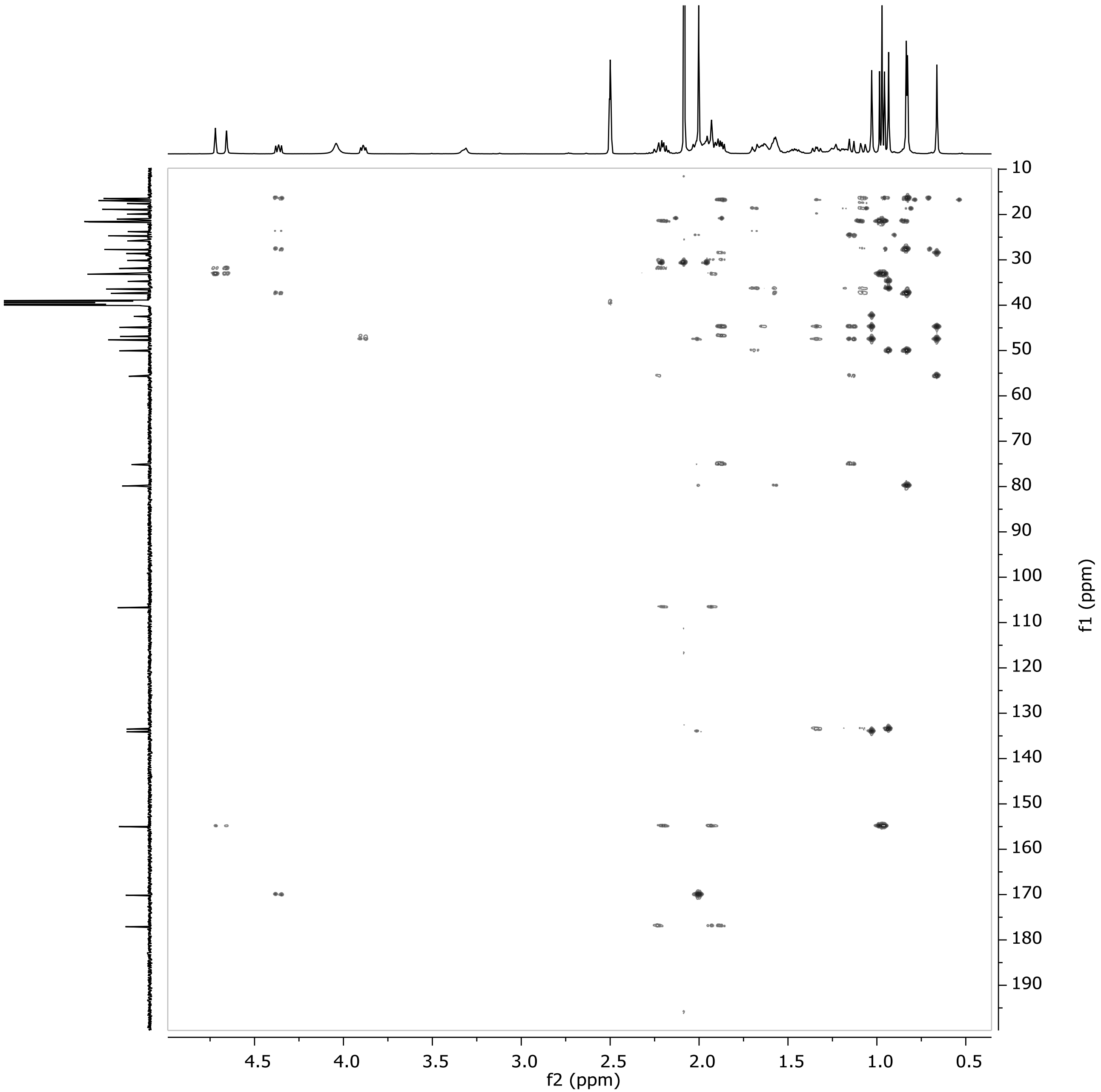

Figure S22. HMBC spectrum of **3** in DMSO-d_6 at 500 MHz.

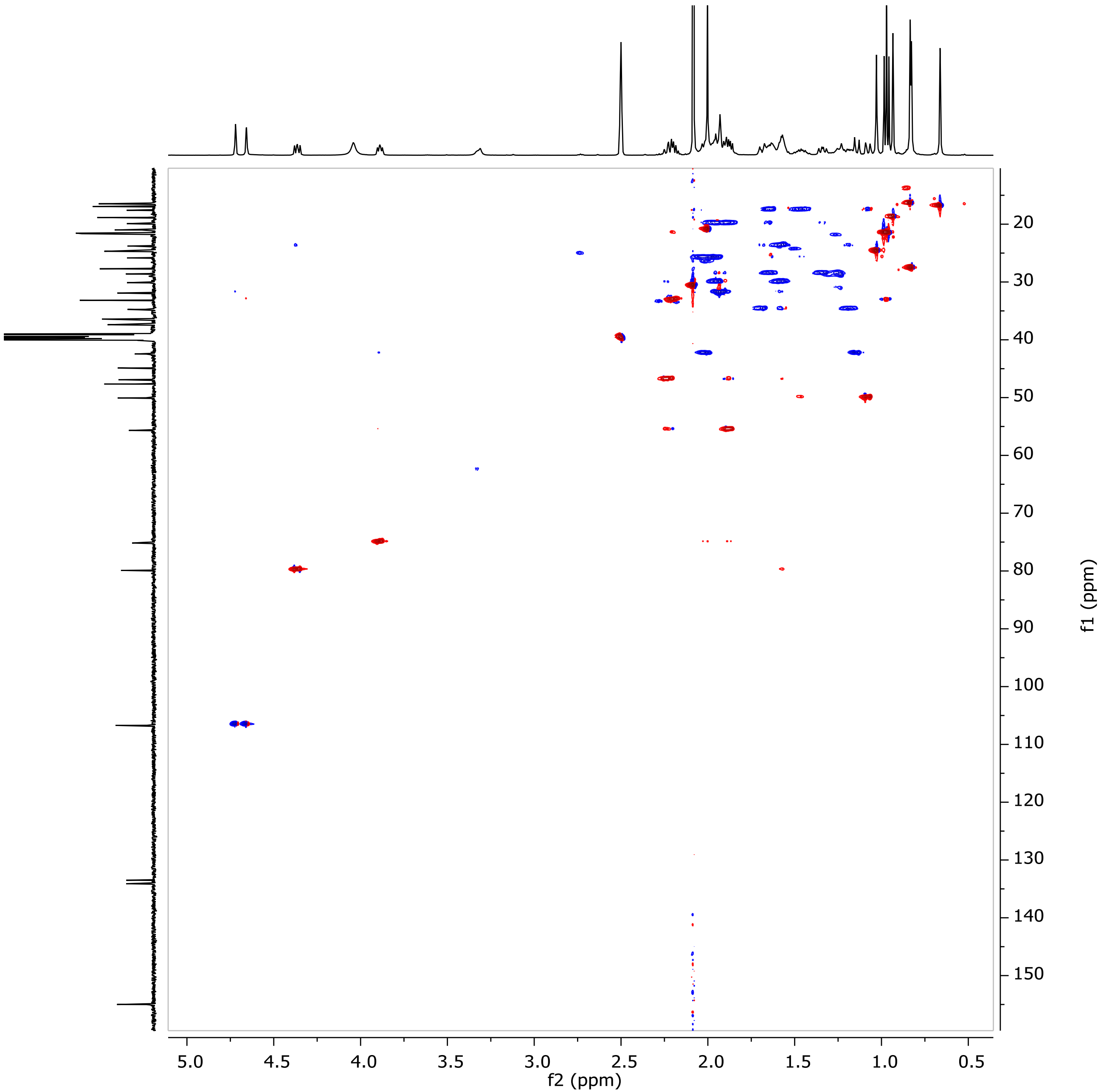

Figure S23. HSQC spectrum of **3** in DMSO-d_6 at 500 MHz.

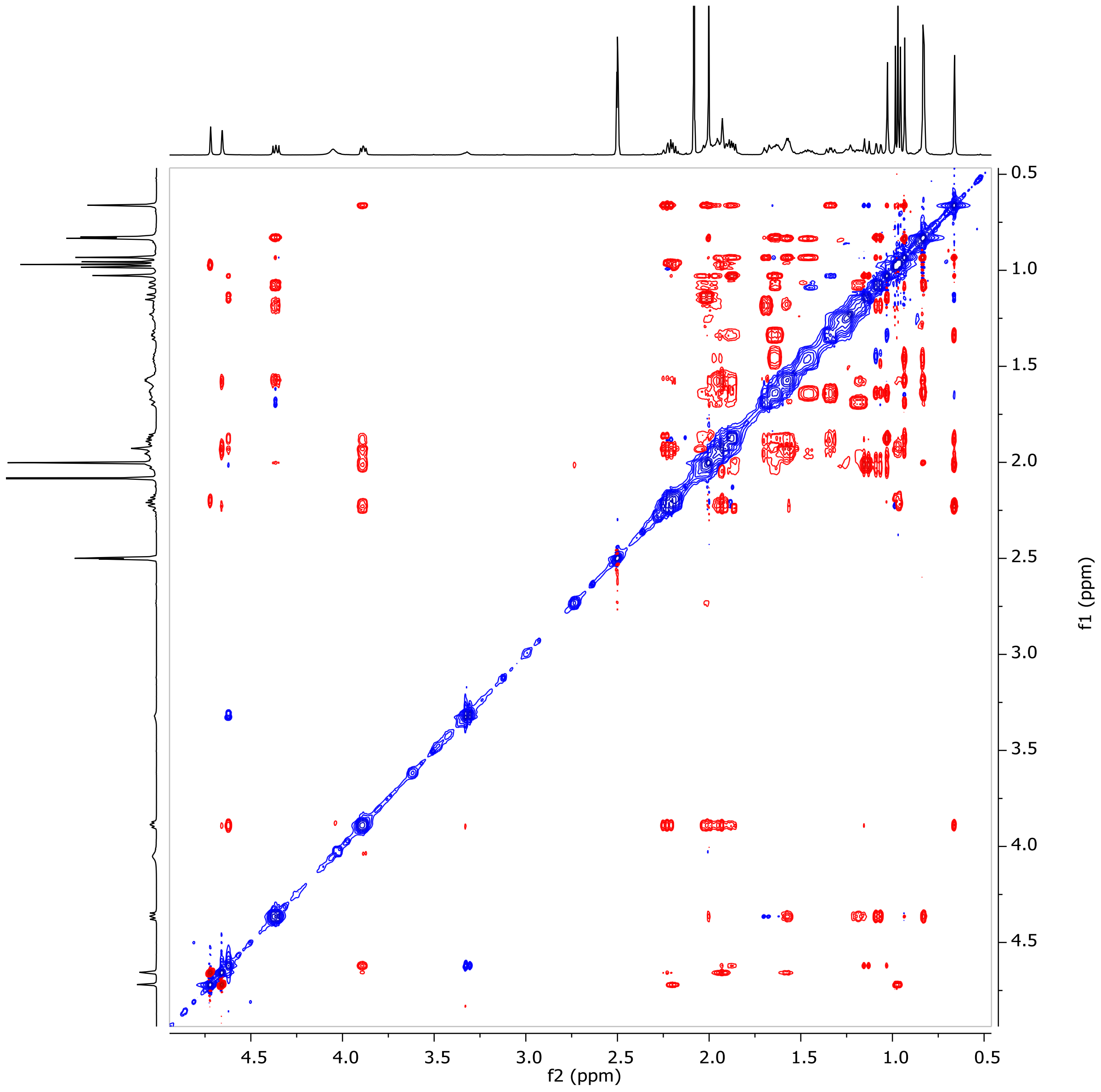

Figure S24. ROESY spectrum of **3** in DMSO-d_6 at 500 MHz.

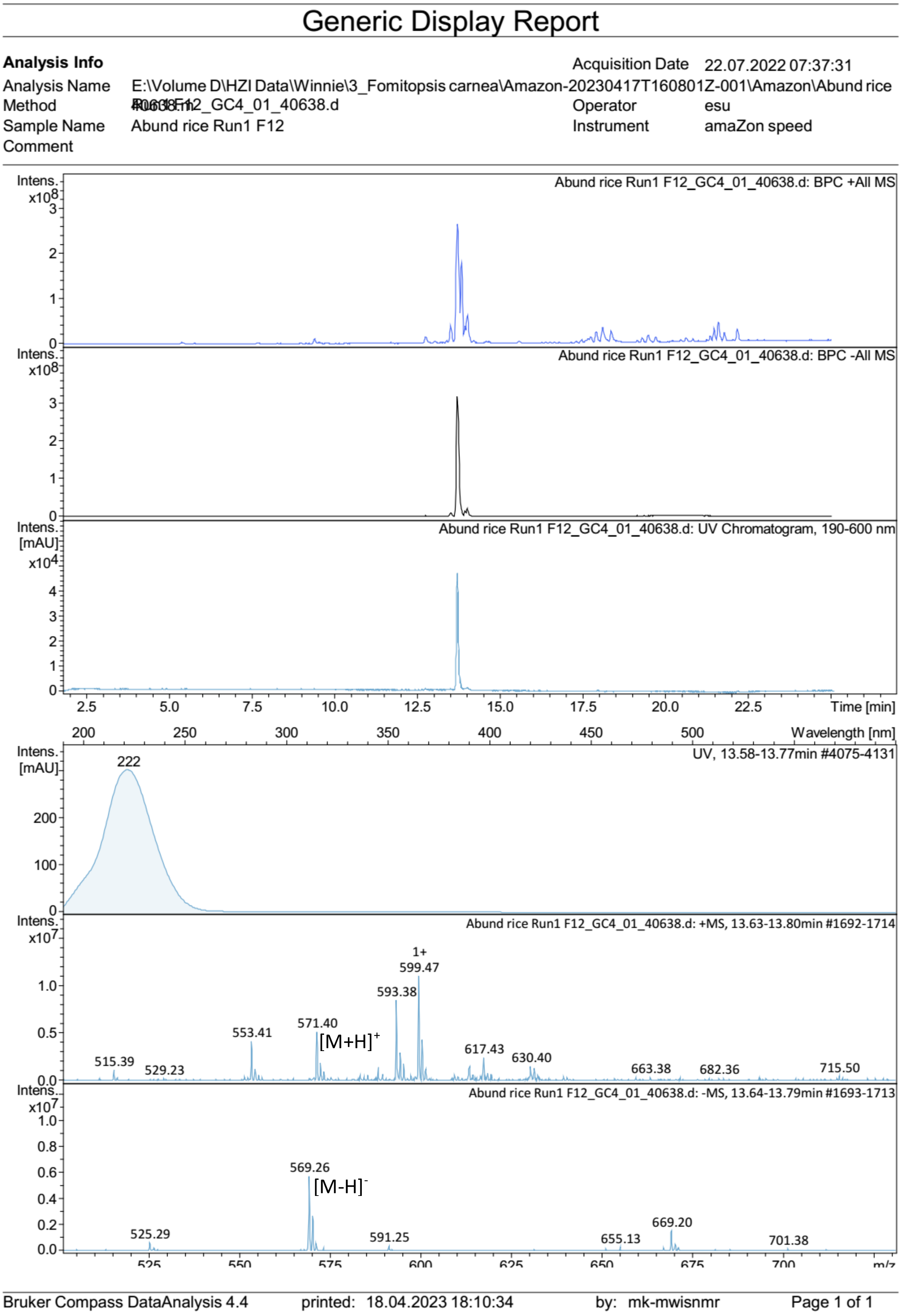

Figure S25. LRESIMS of **4**.

Generic Display Report

Analysis Info

Analysis Name	E:\Volume D\HZI Data\Winnie\3_Fomitopsis	Acquisition Date	15.11.2022 12:51:29
Method	[illegible]	Operator	[illegible]
Sample Name	Abundisporus_495 R1F12	Instrument	maXis
Comment	Screening01<br>Waters Acquity UPLC BEH C_18 1,7um 2.1x50mm		

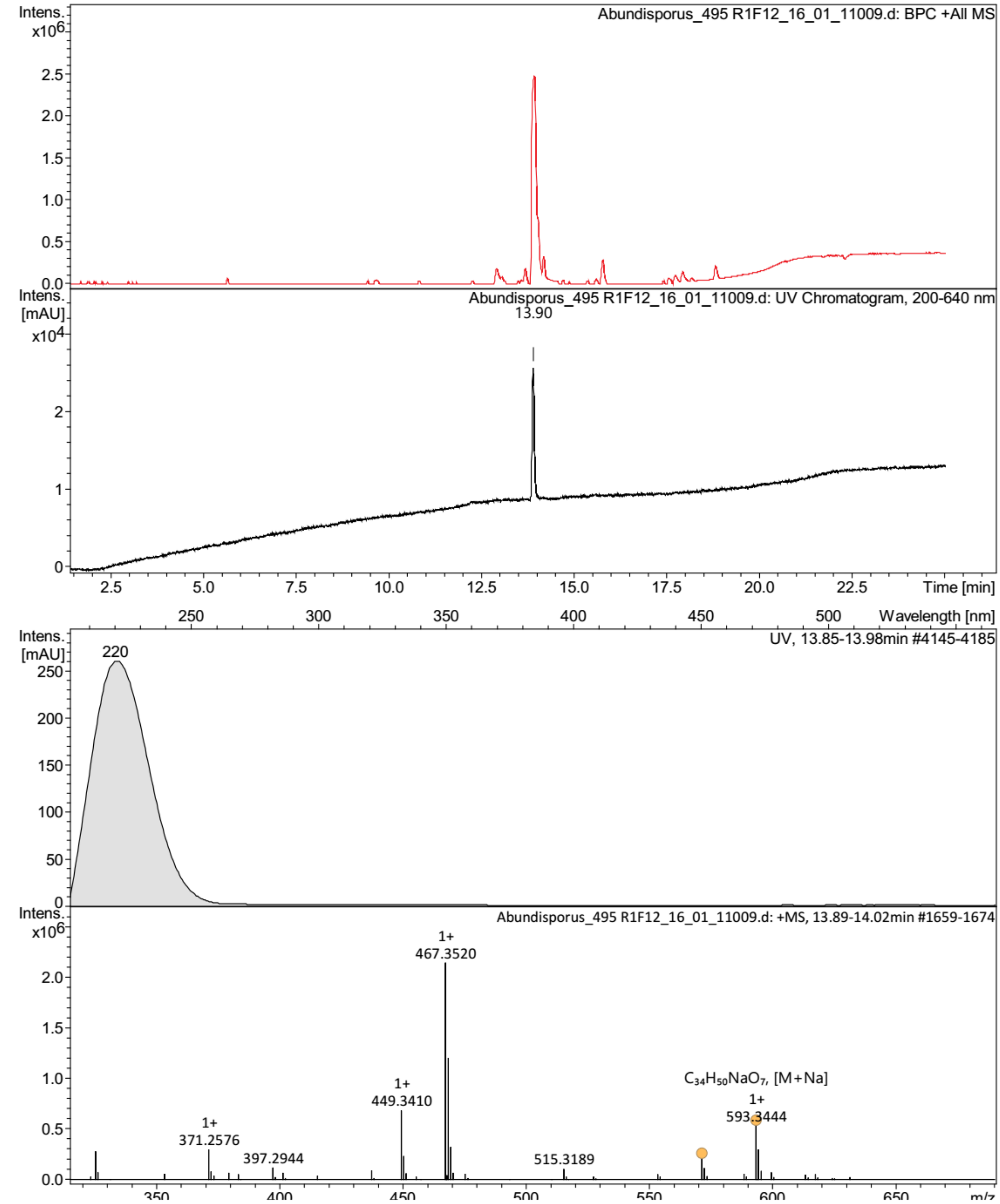

Bruker Compass DataAnalysis 4.4 printed: 18.04.2023 18:01:22 by: mk-mwisnmr Page 1 of 1

Figure S26. HRESIMS of **4**.

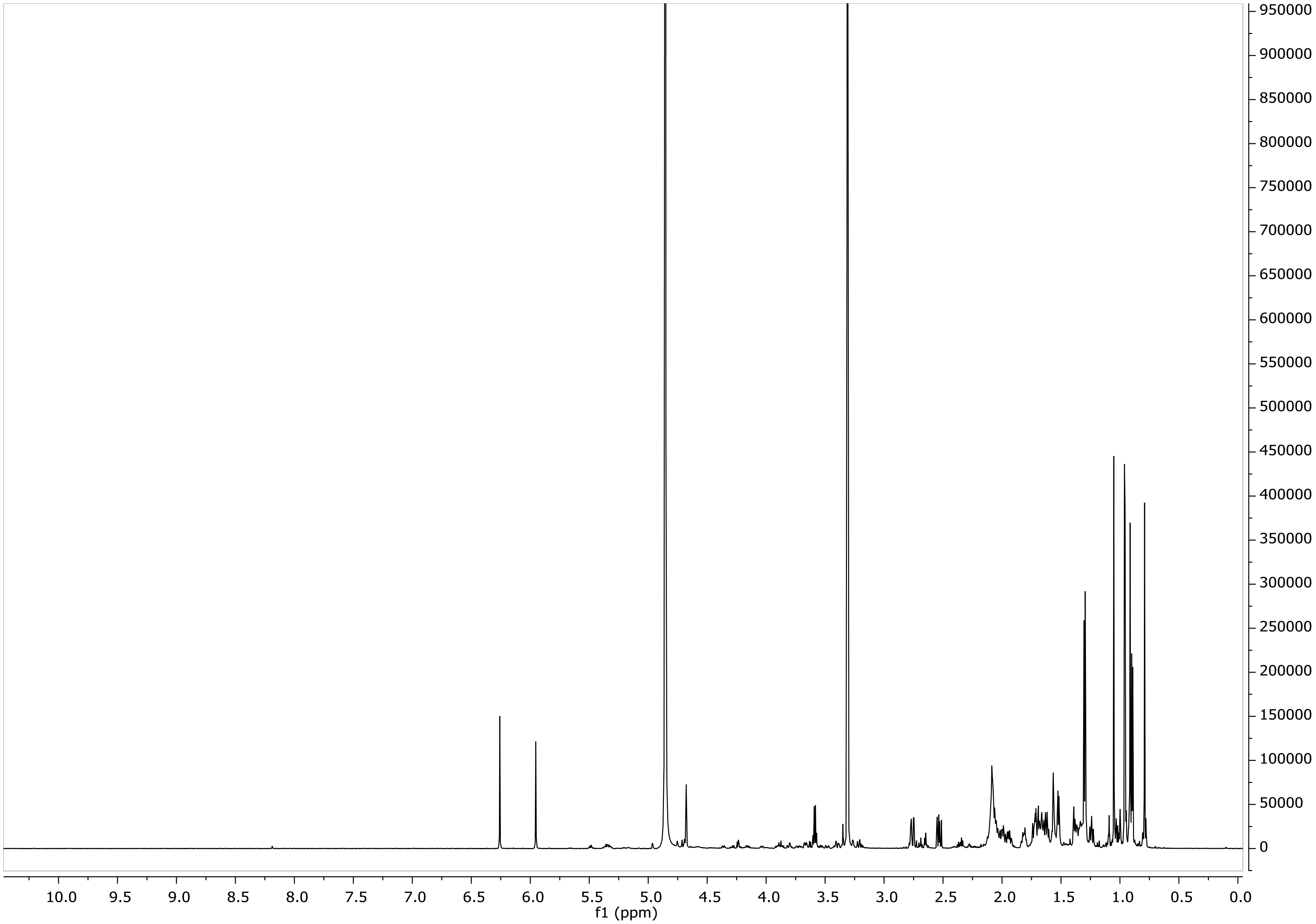

Figure S27. ^{1}H NMR spectrum of **4** in methanol-d_4 at 700 MHz.

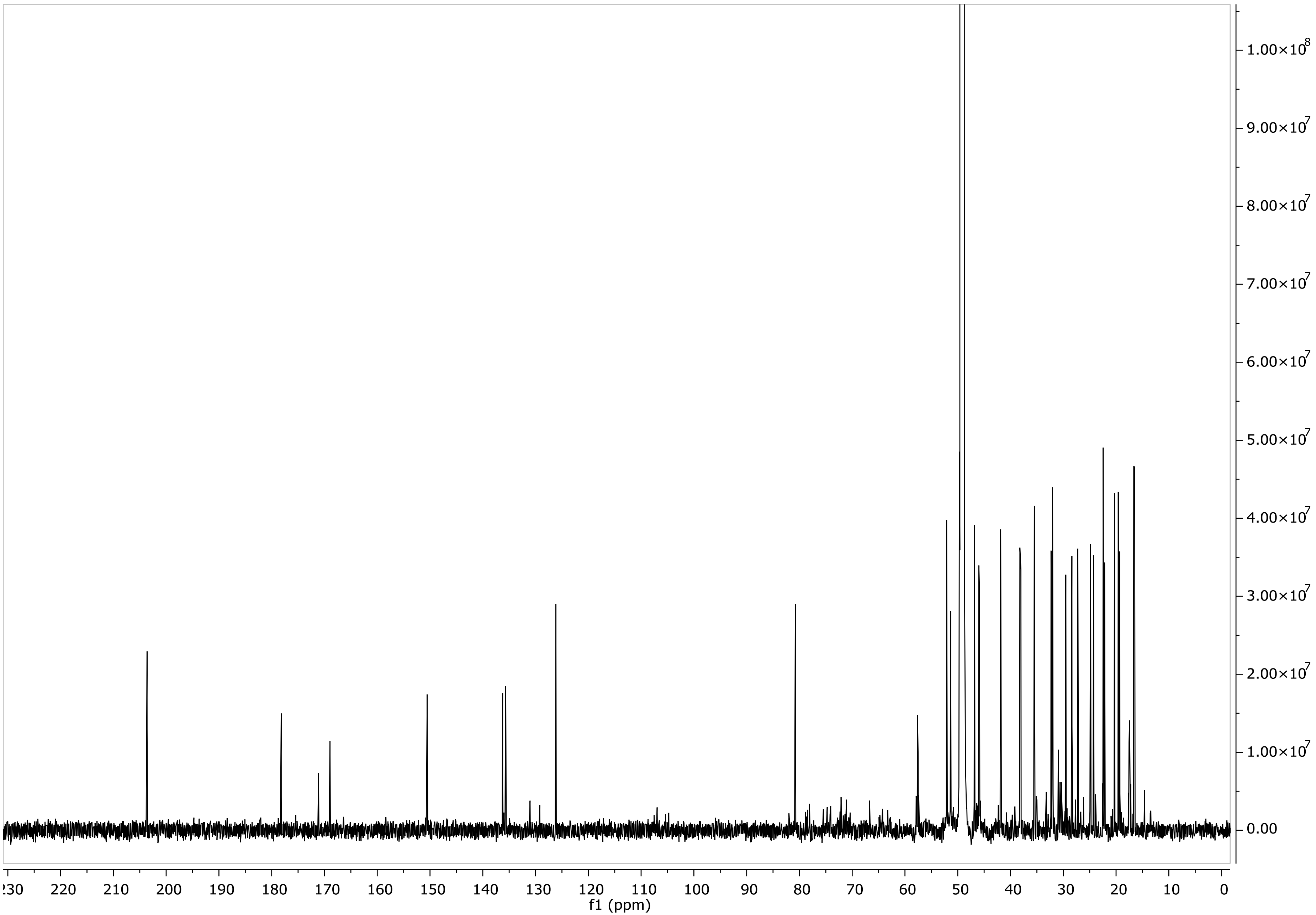

Figure S28. ^{13}C NMR spectrum of **4** in methanol-d_4 at 175 MHz.

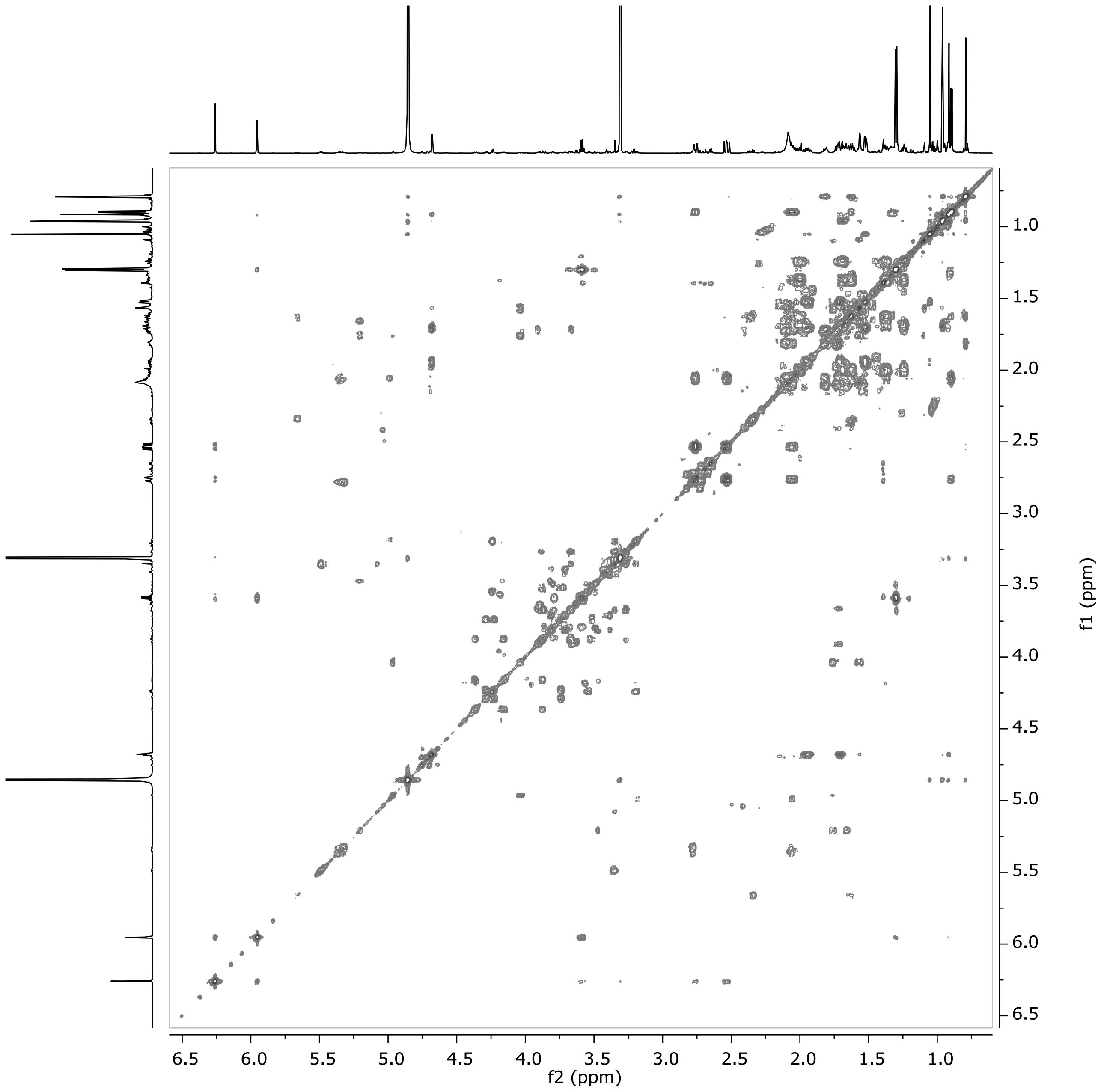

Figure S29. ^{1}H,^{1}H COSY spectrum of **4** in methanol-d_4 at 700 MHz.

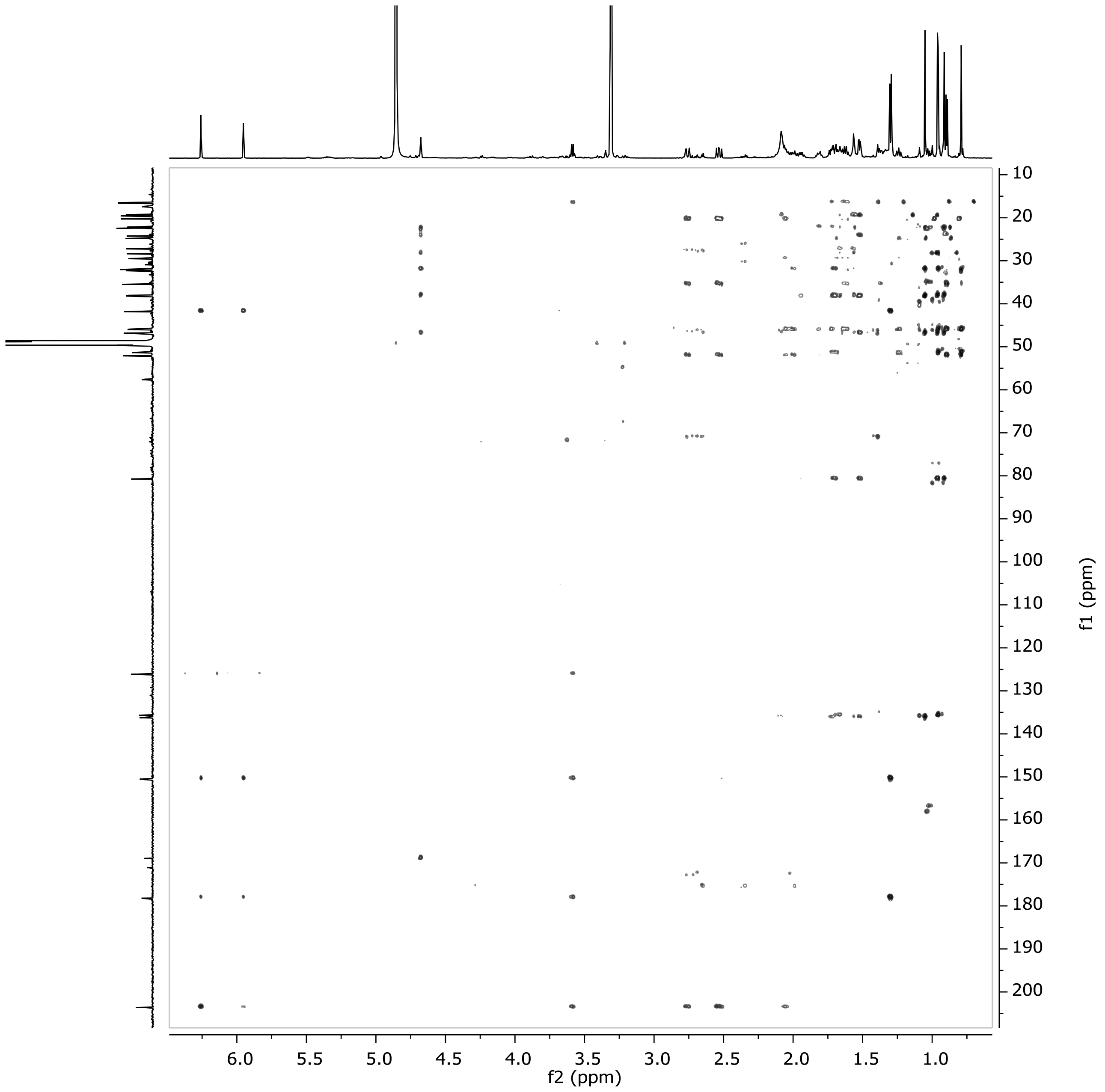

Figure S30. HMBC spectrum of **4** in methanol-d_4 at 700 MHz.

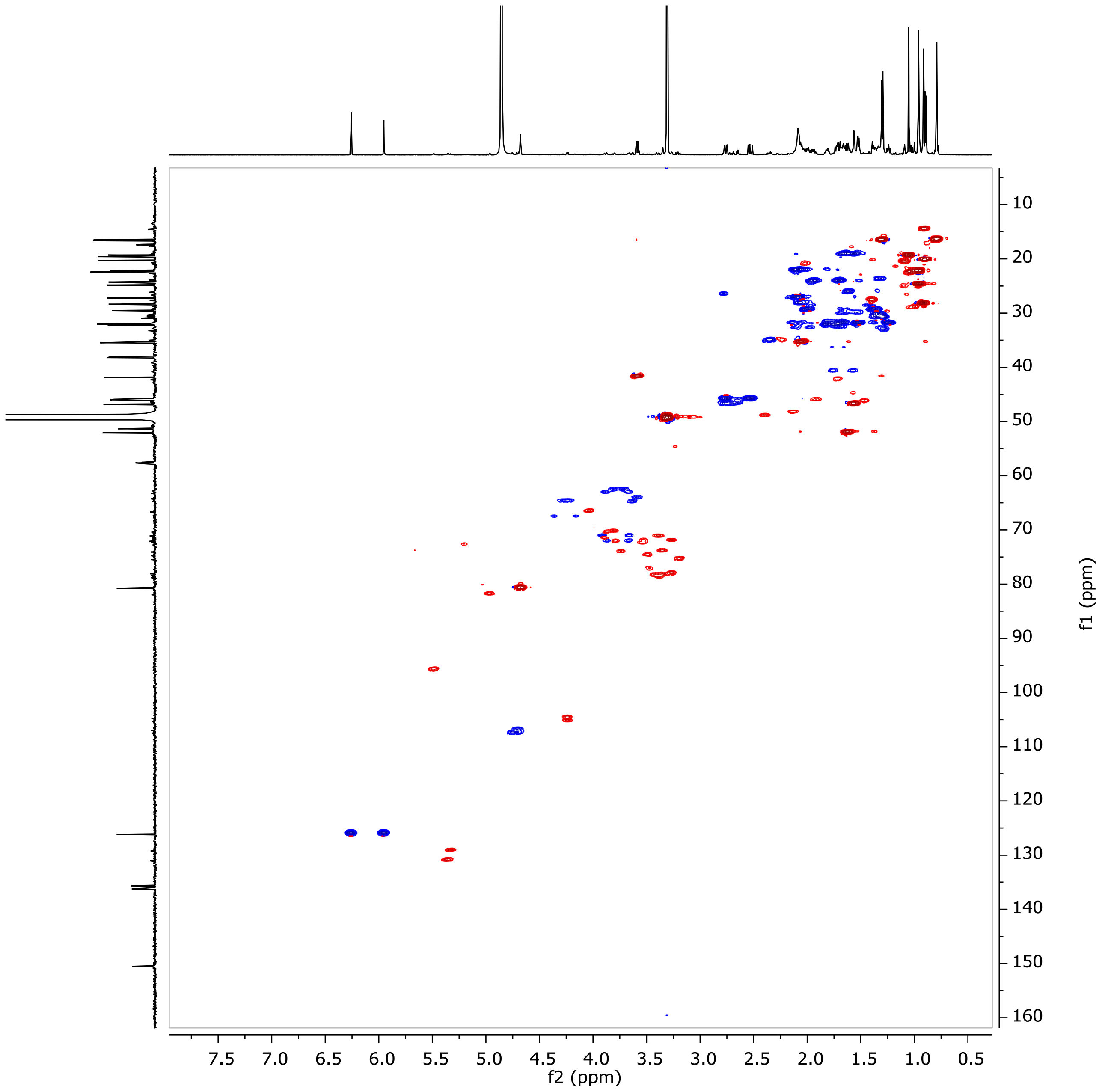

Figure S31. HSQC spectrum of **4** in methanol-d_4 at 700 MHz.

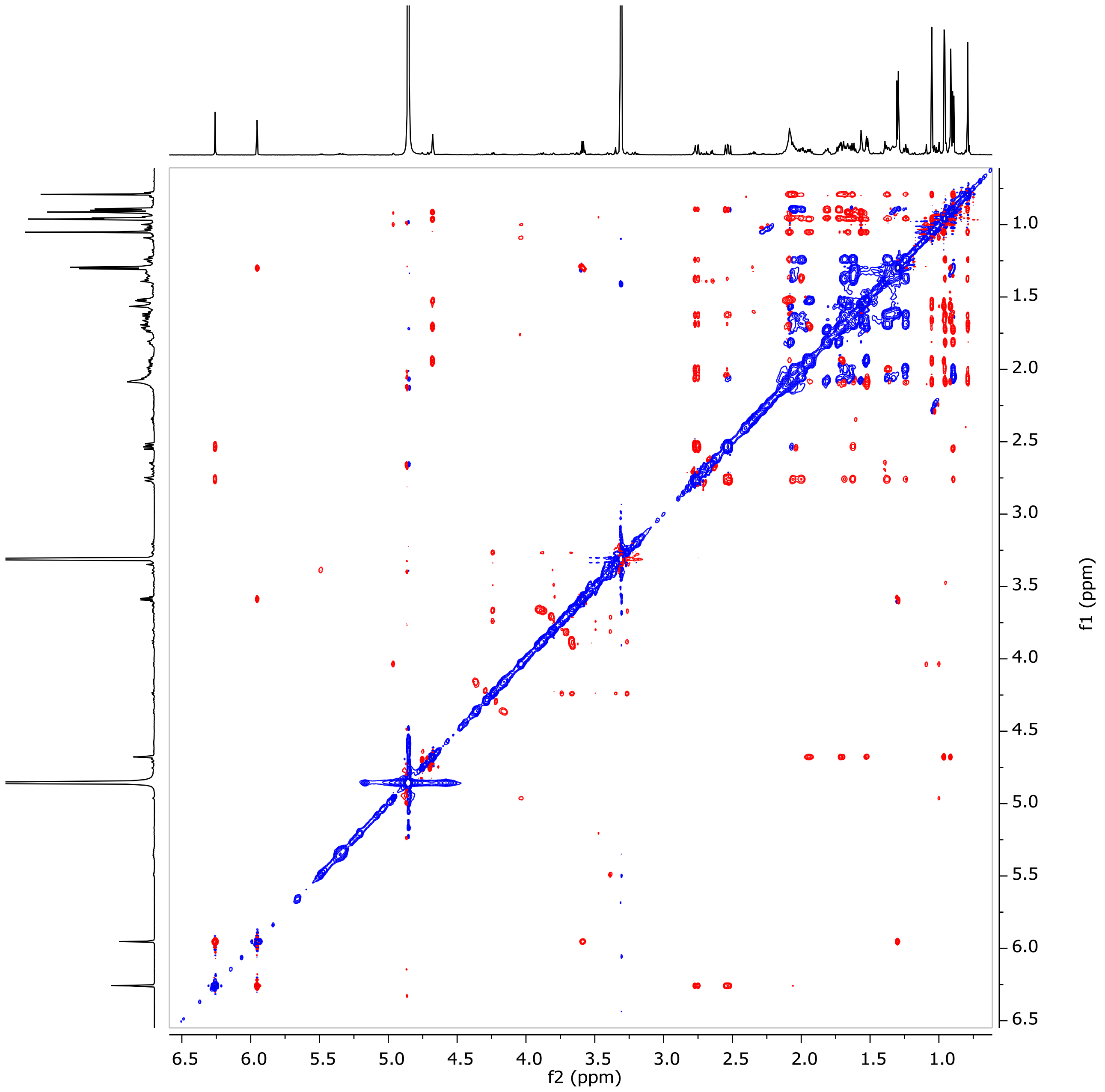

Figure S32. ROESY spectrum of **4** in methanol-d_4 at 700 MHz.
